# Systematics of *Ecnomiohyla tuberculosa* with the description of a new species and comments on the taxonomy of *Trachycephalus typhonius* (Anura, Hylidae)

**DOI:** 10.3897/zookeys.630.9298

**Published:** 2016-11-09

**Authors:** Santiago R. Ron, Pablo J. Venegas, H. Mauricio Ortega-Andrade, Giussepe Gagliardi-Urrutia, Patricia E. Salerno

**Affiliations:** 1 Museo de Zoología, Escuela de Biología, Pontificia Universidad Católica del Ecuador, Av. 12 de Octubre y Roca, Aptdo. 17-01-2184, Quito, Ecuador; 2 División de Herpetología-Centro de Ornitología y Biodiversidad (CORBIDI), Santa Rita N˚105 Of. 202, Urb. Huertos de San Antonio, Surco, Lima, Perú; 3 Laboratorio de Biogeografía, Red de Biología Evolutiva, Instituto de Ecología A.C., Carretera antigua a Coatepec 351, El Haya, CP 91070, Xalapa, Veracruz, México; 4 Programa de Investigación en Biodiversidad Amazónica, Instituto de Investigaciones de la Amazonia Peruana (IIAP), Av. Quiñones Km 2.5, Iquitos, Perú; 5 Current Address: Laboratório de Sistemática de Vertebrados, Pontifícia Universidade Católica do Rio Grande do Sul - PUCRS. Av. Ipiranga, 6681, Porto Alegre, RS 90619-900, Brazil; 6 Department of Biology, Colorado State University, 1878 Campus Delivery, Fort Collins, CO 80523, USA; 7 Current Address: IKIAM, Universidad Regional Amazónica, km 7 vía Muyuna, Tena, Ecuador; 8 Museo Ecuatoriano de Ciencias Naturales, Sección de Vertebrados, División de Herpetología, calle Rumipamba 341 y Av. de los Shyris, Quito, Ecuador

**Keywords:** Advertisement call, Amazon basin, biodiversity, Ecuador, Lophiohylini, Peru, phylogeny, Tepuihyla

## Abstract

*Ecnomiohyla tuberculosa* is an Amazonian hylid of uncertain phylogenetic position. Herein DNA sequences of mitochondrial and nuclear genes are used to determine its phylogenetic relationships. New sequences and external morphology of *Trachycephalus typhonius* are also analyzed to assess the status of Ecuadorian and Peruvian populations. The phylogeny shows unequivocally that *Ecnomiohyla tuberculosa* is nested within the genus *Tepuihyla*, tribe Lophiohylini. This position was unexpected because the remaining species of *Ecnomiohyla* belong to the tribe Hylini. To solve the paraphyly of the genus *Ecnomiohyla*, *Ecnomiohyla tuberculosa* is transferred to the genus *Tepuihyla*. Comparisons of DNA sequences, external morphology, and advertisement calls between populations of *Ecnomiohyla tuberculosa* from Ecuador and Peru indicate that the Peruvian population represents an undescribed species. The new species is described and a species account is provided for *Ecnomiohyla tuberculosa*. *Trachycephalus typhonius* is paraphyletic relative to *Trachycephalus cunauaru*, *Trachycephalus hadroceps*, and *Trachycephalus resinifictrix*. The phylogenetic position of populations from western Ecuador indicates that they represent a species separate from *Trachycephalus typhonius* *sensu stricto*. We resurrect the name *Hyla quadrangulum* (*Trachycephalus quadrangulum* **comb. n.**) for those populations. Amazonian populations of “*Trachycephalus typhonius*” from Ecuador and Peru are genetically and morphologically distinct from *Trachycephalus typhonius* *sensu stricto* and are conspecific with the holotype of *Hyla macrotis*. Therefore, we also resurrect *Hyla macrotis*, a decision that results in *Trachycephalus macrotis* **comb. n.**

## Introduction

Fringe-limbed frogs, genus *Ecnomiohyla* Faivovich et al. 2005, are a group of 14 species distributed in Central America, the Chocó region of Colombia and Ecuador and the Amazon Basin (Batista et al. 2014). Their morphology is distinctive and characterized by large size, proportionally enlarged hands and feet, extensive webbing between fingers, moss-like dorsal coloration, and dermal fringes on the limbs (Batista et al. 2014; Savage and Kubicki 2010). These canopy dwellers that breed on phytotelmata and are rarely found in the lower forest strata (Batista et al. 2014; Mendelson et al. 2008) are scarce in scientific collections with few specimens known for some species.

One of the most enigmatic members of the genus is *Ecnomiohyla tuberculosa* (Boulenger 1882), a species only known from the holotype until the work of Duellman (1974). Its occurrence has been documented at scattered localities along the upper Amazon basin of Brazil, Colombia, Ecuador and Peru (Frost 2014). *Ecnomiohyla tuberculosa* was grouped with other species of *Ecnomiohyla* by Firschein and Smith (1956) and Duellman (1961; 1970) under the “*Hyla tuberculosa*” group. Based on those assignments, Faivovich et al. (2005) included it tentatively in the genus *Ecnomiohyla*. Mendelson et al. (2008) and Savage and Kubicki (2010), based on morphological characters, suggested that *Ecnomiohyla tuberculosa* was not a member of *Ecnomiohyla*. Neither publication proposed an alternative phylogenetic position for *Ecnomiohyla tuberculosa* and they considered it *insertae sedis*. Nevertheless, Batista et al. (2014) maintained the binomial *Ecnomiohyla tuberculosa* and noted that additional information was needed to determine its generic position. Recently, Duellman et al. (2016) placed *Ecnomiohyla tuberculosa* in the genus *Hypsiboas*. We do not follow that assignment because it lacked justification.

Another enigmatic Neotropical hylid is *Trachycephalus typhonius* (Linnaeus 1758). Its identity was recently defined by Lavilla et al. (2010). Previously this species was referred as “*Trachycephalus venulosus*” or “*Phrynohyas venulosa*” (e.g., Duellman 1970; 1971; Faivovich et al. 2005). According to its current definition, *Trachycephalus typhonius* is distributed from southern México to northern Argentina and includes populations in the Guianan region, Amazon Basin, and Chocó (Lavilla et al. 2010). Several authors have reported considerable morphological variation that suggests that it may represent a species complex (e.g., Savage 2002). The existence of at least two species is also suggested by its biogeography. *Trachycephalus typhonius* occurs in the lowlands east and west of the Andes of Ecuador, an unusual pattern only seen in 3 out of 174 species of amphibians: *Hypsiboas boans*, *Rhinella marina* and *Trachycephalus typhonius*. Because the Andes are a formidable barrier to dispersal, this distribution is likely a artifact of incorrect delimitation of species boundaries (dos Santos et al. 2015). The existence of separate species on both sides of the Andes in *Trachycephalus typhonius* is supported by unambiguous morphological differences between both distribution ranges (Ron and Read 2011). Genetic data could conclusively define the status of trans-Andean populations of *Trachycephalus typhonius*.

Fieldwork in Ecuador and Peru has resulted in the collection of additional specimens and genetic samples of *Ecnomiohyla tuberculosa* and *Trachycephalus typhonius*. Based on new specimens of *Ecnomiohyla tuberculosa*, we infer its phylogenetic position and also describe its calling behavior. We show that a distinctive population from Amazonian Peru represents an undescribed species, which we describe here. Finally, we take advantage of the new phylogenetic analysis to include samples of *Trachycephalus typhonius* from Ecuador and Peru and determine their taxonomic status. We show that those populations represent two valid species currently considered junior synonyms of *Trachycephalus typhonius*.

## Materials and methods

### Nomenclature

Generic names follow Faivovich et al. (2005) and AmphibiaWeb (2016).

### DNA extraction amplification and sequencing

DNA was extracted from muscle or liver tissue preserved in 95% ethanol or tissue storage buffer, using standard phenol–chloroform extraction protocols (Sambrook et al. 1989). We used a polymerase chain reaction (PCR) to amplify DNA fragments for mitochondrial genes 12S rRNA (12S), Cytochrome Oxidase sub-unit I (COI), two overlapping fragments for the last ~320 bp of 16S rRNA (16S), NADH dehydrogenase subunit 1 (ND1) and adjacent tRNAs (tRNA^Leu^, tRNA^Ile^ and tRNA^Gln^), and the nuclear gene POMC, using the primers listed in Goebel et al. (1999), Moen and Wiens (2009) and Wiens et al. (2005). PCR amplification was performed under standard protocols and sequenced by the Macrogen Sequencing Team (Macrogen Inc., Seoul, Korea). The combined DNA matrix had up to 3792 bp.

The newly generated DNA sequences are available on GenBank under accession numbers listed on Table 1. To optimize taxon sampling within Hylidae, we blasted our *Ecnomiohyla tuberculosa* 12S and ND1 sequences to the GenBank database (blastn procedure). These searches shown unequivocally that the most similar samples belonged to the genera *Osteocephalus* and *Tepuihyla*, tribe Lophiohylini. For example, a blastn for the ND1 sequence of *Ecnomiohyla tuberculosa* QCAZ 53542 retrieved 50 most similar sequences with identity values ranging from 82.7% to 89.1%. All 50 sequences belonged to the Lophiohylini tribe (*Corythomantis*, *Osteocephalus*, *Osteopilus*, *Tepuihyla*, and *Trachycephalus*). To confirm the close relationships between *Ecnomiohyla tuberculosa* and *Tepuihyla*, we carried out an additional exploratory analysis based on Pyron (2014) matrix for the genes 12S and 16S. We included our sequences as well as those from all species of the tribes Hylini (including *Ecnomiohyla*) and Lophiohylini from Pyron (2014) matrix. We also included several species of *Agalychnis*, *Litoria* and *Phyllomedusa* as outgroups. The final matrix had 169 terminals. A maximum likelihood phylogenetic analysis using GARLI 2.0 (Zwickl 2006) confirmed with strong support that *Ecnomiohyla tuberculosa* is not closely related to other *Ecnomiohyla*. Instead, it is a member of the tribe Lophiohylini and is nested within *Tepuihyla*. The phylogeny based on Pyron (2014) matrix is available as Suppl. material 1.

**Table 1.** New sequences generated for the phylogenetic analysis.

Voucher	Species	12S	16S-ND1	COI	POMC	GenSeq Nomenclature
CORBIDI 12513	*Tepuihyla shushupe*	KY013372	KY013419	KY013396	-	genseq-1
QCAZ 52855	*Tepuihyla tuberculosa*	KY013373	KY013417	-	-	genseq-4
QCAZ 53147	*Tepuihyla tuberculosa*	KY013374	KY013416	-	-	genseq-4
QCAZ 53542	*Tepuihyla tuberculosa*	KY013375	KY013418	KY013415	-	genseq-4
105BM	*Trachycephalus coriaceus*	KY013376	KY013420	-	-	-
QCAZ 19305	*Trachycephalus cunauaru*	KY013388	KY013440	KY013395	KY013462	genseq-4
QCAZ 20808	*Trachycephalus cunauaru*	-	KY013443	KY013398	KY013465	genseq-4
QCAZ 20809	*Trachycephalus cunauaru*	KY013389	KY013441	-	KY013463	genseq-4
QCAZ 46436	*Trachycephalus cunauaru*	KY013390	KY013442	KY013413	KY013464	genseq-4
CORBIDI 9556	*Trachycephalus macrotis*	KY013392	-	-	-	genseq-4
CORBIDI 9559	*Trachycephalus macrotis*	KY013393	-	-	-	genseq-4
CORBIDI 9560	*Trachycephalus macrotis*	KY013394	KY013430	-	-	genseq-4
QCAZ 18198	*Trachycephalus macrotis*	-	KY013428	KY013397	KY013451	genseq-4
QCAZ 21283	*Trachycephalus macrotis*	-	KY013421	KY013400	KY013445	genseq-4
QCAZ 23997	*Trachycephalus macrotis*	-	KY013422	-	KY013444	genseq-4
QCAZ 32492	*Trachycephalus macrotis*	-	KY013426	KY013405	KY013449	genseq-4
QCAZ 38075	*Trachycephalus macrotis*	KY013380	KY013427	KY013406	KY013450	genseq-4
QCAZ 38774	*Trachycephalus macrotis*	KY013377	KY013423	KY013407	KY013446	genseq-4
QCAZ 39565	*Trachycephalus macrotis*	KY013378	KY013424	KY013409	KY013447	genseq-4
QCAZ 43017	*Trachycephalus macrotis*	KY013379	KY013425	KY013412	KY013448	genseq-4
QCAZ 47319	*Trachycephalus macrotis*	KY013381	KY013429	KY013414	KY013452	genseq-4
QCAZ 21282	*Trachycephalus quadrangulum*	-	KY013439	KY013399	KY013461	genseq-4
QCAZ 23472	*Trachycephalus quadrangulum*	-	KY013438	KY013401	KY013460	genseq-4
QCAZ 23485	*Trachycephalus quadrangulum*	KY013387	KY013437	KY013402	KY013459	genseq-4
QCAZ 28530	*Trachycephalus quadrangulum*	KY013383	KY013432	KY013403	KY013454	genseq-4
QCAZ 31271	*Trachycephalus quadrangulum*	KY013382	KY013431	KY013404	KY013453	genseq-4
QCAZ 38205	*Trachycephalus quadrangulum*	-	KY013434	-	KY013456	genseq-4
QCAZ 39360	*Trachycephalus quadrangulum*	KY013385	KY013435	KY013408	KY013457	genseq-4
QCAZ 39782	*Trachycephalus quadrangulum*	KY013386	KY013436	KY013410	KY013458	genseq-4
QCAZ 40219	*Trachycephalus quadrangulum*	KY013384	KY013433	KY013411	KY013455	genseq-4
163MC	*Trachycephalus typhonius*	KY013391	-	-		-

For final phylogenetic analysis we included GenBank sequences from all available species of Lophiohylini as well as sequences from representative species of all other tribes within Hylinae (Cophomantini, Dendropsophini, and Hylini). GenBank sequences were originally published by Darst and Cannatella (2004), Faivovich et al. (2005), Salducci et al. (2005), Wiens et al. (2005), Moen and Wiens (2009), Moravec et al. (2009), Salerno et al. (2012), Jungfer et al. (2013), and Salerno et al. (2015). Samples of *Phyllomedusinae* were included as outgroups (*Agalychnis spurrelli*, *Phyllomedusa tomopterna*, and *Phyllomedusa perinesos*). We also added new sequences from 25 individuals of Ecuadorian and Peruvian *Trachycephalus* to assess their taxonomic status.

Preliminary sequence alignment was done with MAFFT 7.2 software with the L-INS-i algorithm (Katoh and Standley 2013). All sequences in the matrix were visually examined and translated in MESQUITE (version 3.01; Maddison and Maddison 2014). The matrix was partitioned to allow independent inferences of models of evolution by gene and by codon position in coding genes. We used software PARTITIONFINDER v. 1.1.1 (Lanfear et al. 2012) to simultaneously estimate both the best-fit model for each partition and the best partition strategy for our data.

### Phylogeny

Phylogenetic trees were obtained using maximum likelihood and Bayesian inference. Maximum likelihood searches were carried out with software GARLI 2.0 (Zwickl 2006). We made two independent searches with 10 replicates each. The first search started with random trees and the second with stepwise addition trees. We modified the settings for the number of generations without topology improvement required for termination (genthreshfortopoterm = 200000) and the maximum range for localized SPR topology changes (limsprrange = 10) to increase exhaustiveness of the tree space search. Other settings were set on default values. Node support was assessed with 200 pseudoreplicate non-parametric bootstraps (npb), configured with the same settings of the full search, but with three replicates per run.

Bayesian inference was performed with MARBAYES 3.2.1 (Ronquist et al. 2012). The analysis consisted of four parallel runs of the Metropolis-coupled Monte Carlo Markov chain for 5 × 10^6^ generations; each run had six chains with a temperature of 0.08. We used TRACER 1.6 (Rambaut et al. 2014) to assess convergence and stationarity of the runs and to obtain effective sample sizes (ESS) for all model parameters. The search was considered finished when all ESS were > 200. Each run was sampled every 1000 generations. The first 25% of the samples was discarded as “burn-in”, using the remaining samples to estimate the Bayesian tree, posterior probabilities (pp) and other model parameters.

### Morphology

Diagnostic characters and comparisons are based on specimens from the following institutions. Ecuador: Fundación Herpetológica Gustavo Orcés, Quito (FHGO); Museo de Zoología at Pontificia Universidad Católica del Ecuador, Quito (QCAZ). Peru: Centro de Ornitología y Biodiversidad, Lima (CORBIDI); Colección Referencial de Biodiversidad del Instituto de Investigaciones de la Amazonía Peruana, Iquitos (IIAP). United States of America: American Museum of Natural History, New York, (AMNH); National Museum of Natural History, Washington DC (USNM); Natural History Museum at the University of Kansas, Lawrence (KU). United Kingdom: Natural History Museum, London
(BMNH). Venezuela: Museo de Historia Natural La Salle, Caracas (MHNLS). Our comparisons included the revision of the holotype of *Hyla tuberculosa* (Natural History Museum, London, BMNH 1947.2.13.34) and photographs of the holotype of *Hyla macrotis* (Swedish Museum of Natural History, Stockholm, NRM 1958). Examined specimens are listed as Suppl. material 2. Character definitions follow Duellman (1970) and Luna et al. (2012) for nuptial excrescences. Notation for hand and foot webbing is based on Myers and Duellman (1982). Sex was determined by presence of nuptial pads or vocal slits, and by gonadal inspection. Descriptions of coloration in life are based on digital photographs. Adults were measured with digital calipers for the following eight morphological variables, following Duellman (1970): (1) SVL; (2) head length; (3) head width; (4) tympanum diameter; (5) femur length; (6) tibia length; (7) foot length; and (8) eye diameter. Additionally, we measured the hand length (HAL) from proximal edge of palmar tubercle to tip of third finger for the new species described herein following Batista et al. (2014).

Morphometric analyses were performed based on measurements of adult males (number of specimens in parenthesis): *Ecnomiohyla tuberculosa* (5), *Tepuihyla shushupe* sp. n. (1), *Tepuihyla edelcae* (17), *Tepuihyla obscura* (17), and *Tepuihyla rodriguezi* (14; see Suppl. material 2). We did a log_10_ transformation of the dataset, a common practice in morphometric analyses due to the multiplicative nature of allometric growth and to make measurement variances comparable. A principal components analysis was later performed on the data. We used Principal Component 1 as a proxy for size and its effects, given that most variation is due to size and size effect differences. Other principal components were interpreted as a proxy for shape (Bookstein 1989). We also performed a Discriminant Analysis with species as group priors, in order to determine if they can be diagnosed from each other with morphometric characters.

### Bioacoustics

Calls were analyzed from two adult males, QCAZ 53699 from Juyuintza, Pastaza province, Ecuador (2.110°S, 76.190°S, 200 m of elevation) and CORBIDI 12513 from Ere River, Putumayo Basin, Loreto Department, Peru (1.67903°S, 73.7197°W, 145 m). Male QCAZ 53699 was recorded at night on 22 June 2012 (23.9°C / relative humidity ~86.0%). Male CORBIDI 12513 was recorded at night of 18 October 2012 (21.3°C / relative humidity ~95.8%). Recordings were saved in PCM format, with a sample rate of 48000 Hz and 24-bits. Call variables were measured with RAVEN PRO 1.4 (Charif et al. 2010), under a Hanning function, 2048 DFT sample size and a grid spacing of 23.4 Hz. After a preliminary screening of the sound spectrogram, we clipped values below 90.0 db and applied a filter band-pass between 350 and 2420 Hz, to reduce background noise and facilitate the measurement of the acoustic parameters. Fourteen acoustic parameters (modified from Cocroft and Ryan 1995) were measured to describe the structure of each call: (1) Call duration = time from beginning to end of one call, measured from waveform analyzer screen; (2) Call rate = number of calls per minute; (3) Call interval = time from the end of the call to the beginning of the next call; (4) Call rise time = time from beginning of call to point of maximum amplitude; (5) Notes per call = number of notes per call; (6) Note rate = (number of notes-1)/time from beginning of first note to beginning of last note; (7) Note length = average time from beginning to end of a note; (8) Note rise time = time from beginning of note to point of maximum amplitude; (9) Note shape = note rise time/note length; (10) Frequency band = difference between the upper and lower frequencies measured along the entire call; (11) Fundamental frequency = frequency with the highest energy on the 1st harmonic in the call; (12) Dominant frequency = frequency with the highest energy along the entire call. In order to analyze the modulation of dominant frequency, we divided the call in three sections corresponding to the beginning, the middle and the end of the call; (13) Fundamental frequency ratio = number of notes with the dominant frequency in the first harmonic/total number of notes; and (14) Frequency modulation = (frequency with the most energy at end of call – frequency with the most energy at beginning of call)/call duration; values equal or near 0 represent calls lacking frequency modulation. Recordings are deposited in the Sound Archive of Museo de Zoología of Pontificia Universidad Católica del Ecuador (available at AmphibiaWebEcuador website, http://zoologia.puce.edu.ec/vertebrados/anfibios/).

## Results

### Phylogeny

**Figure 1.**
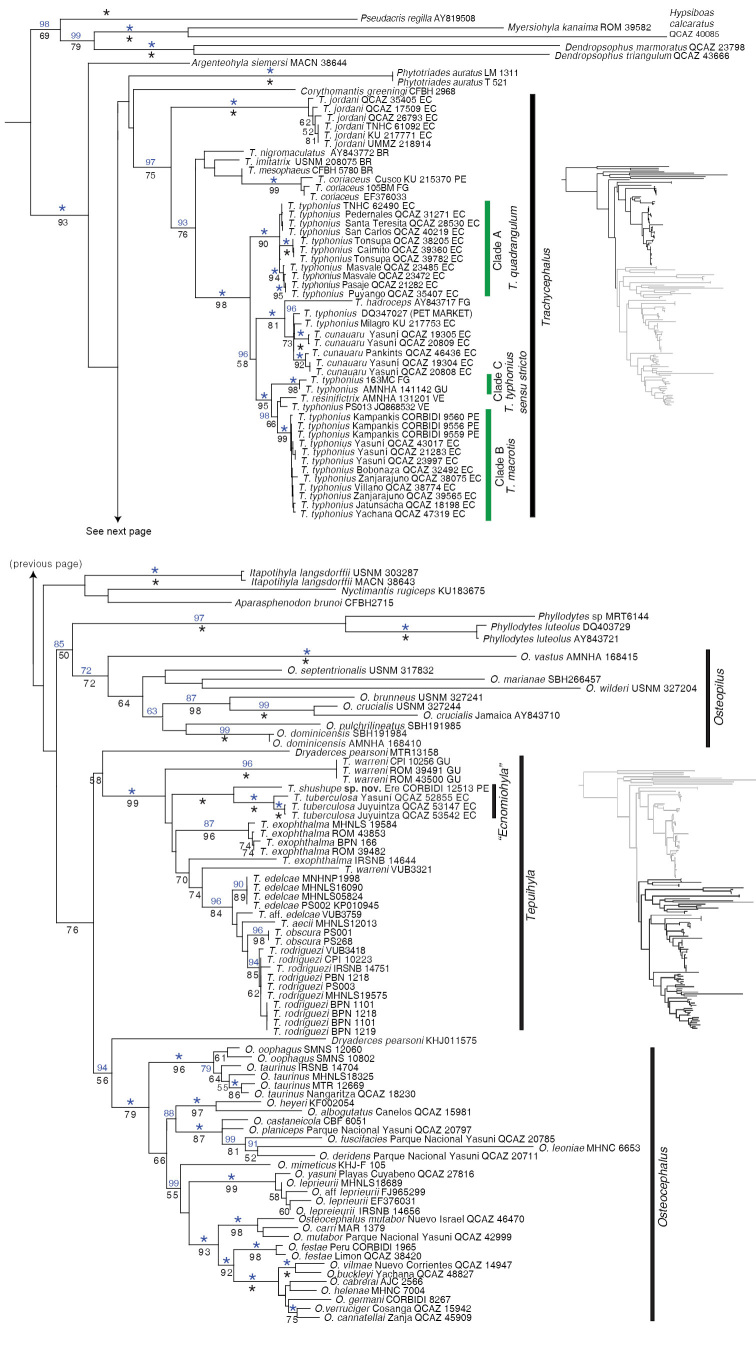
Maximum likelihood phylogram showing the position of *Tepuihyla tuberculosa* comb. n. and *Tepuihyla shushupe* sp. n. within Hylidae. Phylogram derived from analysis of 3792 bp of mitochondrial (gene fragments *12S*, *16S*, *ND1*, *CO1*, *tRNA ^Leu^*, *tRNA ^Ile^*, *tRNA ^Gln^*) and nuclear DNA (POM-C). Voucher no. (or, if unavailable, GenBank accession no.) is shown for each sample. Clade posterior probabilities (pp × 100) resulting from Bayesian Markov chain Monte Carlo searches appear above branches in blue. Non-parametric bootstrap (npb) support values, from 200 pseudoreplicates, are shown below. Asterisks represent values of 100. Outgroups are not shown. Abbreviations are: BR = Brazil, EC = Ecuador, FG = French Guiana, GU = Guyana, PE = Peru, VE = Venezuela

The Maximum Likelihood and the Bayesian analyses yielded similar topologies with differences pertaining only to weakly supported nodes. The phylogeny shows strong support for the inclusion of *Ecnomiohyla tuberculosa* within the tribe Lophiohylini, genus *Tepuihyla* Ayarzagüena et al. 1993 “1992” (Fig. 1B). *Ecnomiohyla tuberculosa* is sister to all species of *Tepuihyla*, except *Tepuihyla warreni* from Ayanganna, Guyana. *Tepuihyla warreni* is polyphyletic because a sample from the Maringma Tepui, Guyana, clusters with *Tepuihyla aecii*, *Tepuihyla edelcae*, *Tepuihyla obscura*, and *Tepuihyla rodriguezi* instead of the other samples of *Tepuihyla warreni* (Fig. 1A). The distance of the type locality of *Tepuihyla warreni* from Ayanganna is approximately 90 km and from Maringma is 15 km. These distances suggest that the Ayanganna population represents an undescribed species.

There is strong support for the tribe Lophiohylini. However, the relationships between basal clades within Lophiohylini are weakly supported. Within *Trachycephalus*, the recently described *Trachycephalus cunauaru* is sister to *Trachycephalus hadroceps*. *Trachycephalus typhonius* (previously referred as “*Trachycephalus venulosus*”) is widely paraphyletic. The new sequences of *Trachycephalus typhonius* from the Ecuadorian Chocó are sister to a clade composed of *Trachycephalus cunauaru*, *Trachycephalus hadroceps*, *Trachycephalus resinifictrix*, *Trachycephalus typhonius* from Guyana, French Guiana, Venezuela, and the upper Amazon basin in Ecuador and Peru. Populations of *Trachycephalus typhonius* from Amazonian Peru and Ecuador are sister to a clade composed of *Trachycephalus resinifictrix* and one sample of *Trachycephalus typhonius* from Venezuela (Fig. 1A).

As in previous phylogenies (e.g., Pyron 2014) we found a clade composed by *Dryaderces* + *Tepuihyla* + *Osteocephalus*. Two samples of *Dryaderces* do not cluster together. This is likely a result of the use of short and non-overlapping sequences between both samples. Therefore, our results do not challenge the monophyly of *Dryaderces*.

### Morphometry

**Figure 2.**
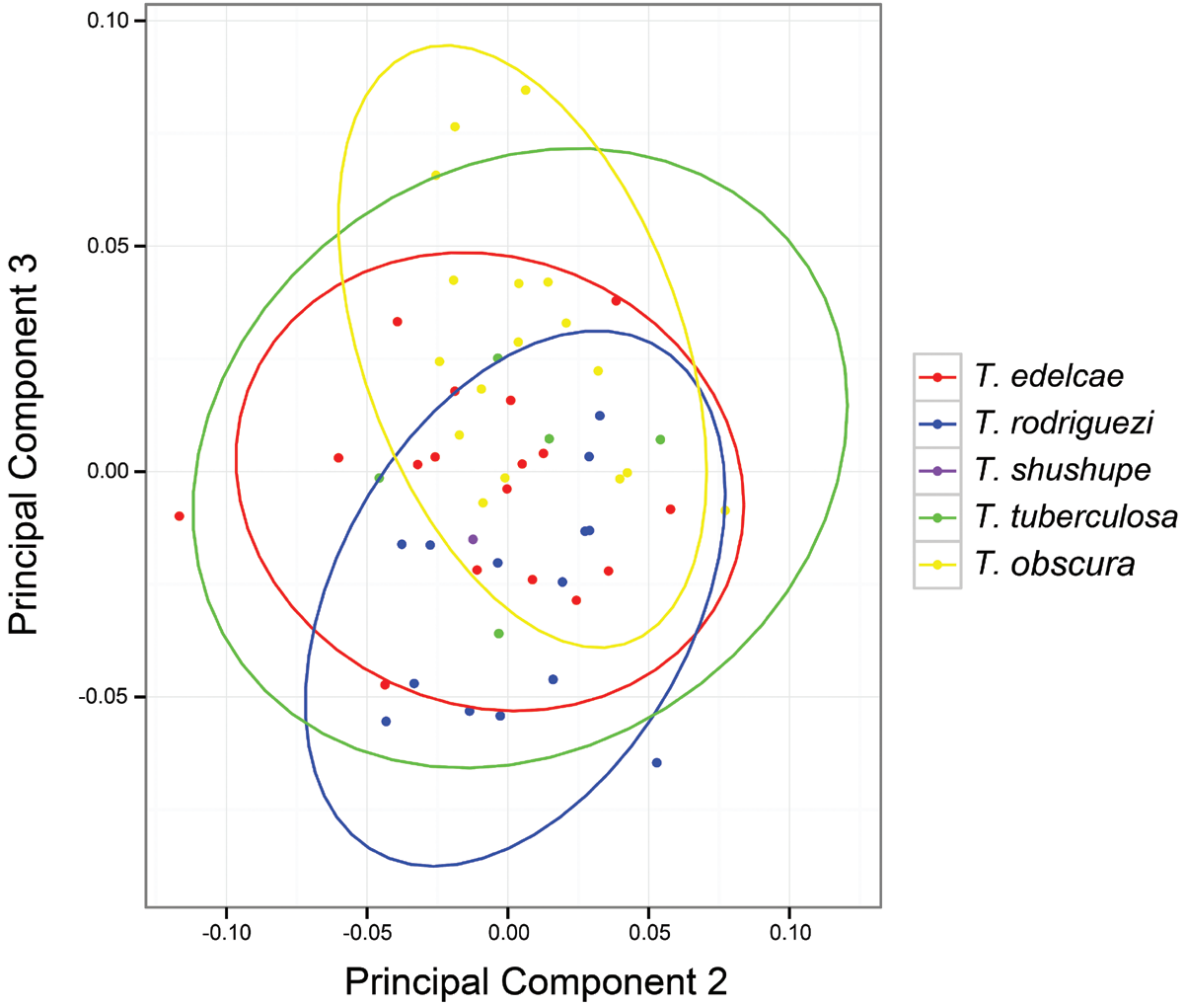
Principal Components 2 and 3 from analysis of eight morphological variables. See Table 2 for character loadings on each component.

The first Principal Component, a proxy for size covariation, explains 97% of the variance and has very similar proportions for all variables (Table 2). The second Principal Component is mostly made up of Eye Diameter, but the variance seems to be higher within rather than among groups (Fig. 2). The third principal component is mainly composed of Tympanum Diameter.

**Table 2.** Character loadings and cumulative proportion of variance for Principal Components. The analysis was based on eight morphometric variables of adult males of *Tepuihyla tuberculosa* comb. n., *Tepuihyla shushupe* sp. n., *Tepuihyla edelcae*, *Tepuihyla obscura*, and *Tepuihyla rodriguezi*. PCs 6–8 not shown. Abbreviations are: SVL = snout-vent length; FOOT = foot length; HL = head length; HW = head width; ED = eye diameter; TD = tympanum diameter; TL = tibia length; FL = femur length.

	PC1	PC2	PC3	PC4	PC5
Cumulative variance	0.978	0.987	0.996	0.997	0.998
SVL	-0.371	0.052	-0.117	0.079	-0.302
FOOT	-0.421	0.221	0.267	-0.413	0.707
HL	-0.329	0.054	0.078	0.417	-0.072
HW	-0.379	0.000	-0.008	0.643	0.204
ED	-0.323	-0.898	0.205	-0.186	-0.082
TD	-0.272	-0.074	-0.912	-0.176	0.137
TL	-0.393	0.229	0.168	0.005	-0.197
FL	-0.316	0.284	0.076	-0.412	-0.545

Morphometric analyses do not show differences in shape among species (Fig. 2). The only morphometric difference pertains to size (Principal Component 1; not shown) as *Ecnomiohyla tuberculosa* is much larger than the other species. The Discriminant Function Analysis graph (not shown) is similar to the PCA in not showing interspecific differences. Group assignment proportions are all very low (between 0.02–0.31), indicating very little power of morphometric discrimination among groups, even when attempting to maximize differences with species/lineage priors.

### Comparisons between populations of *Ecnomiohyla tuberculosa*

The four samples of *Ecnomiohyla tuberculosa* form three well-supported clades (pp/npb = 100). The sample from Ere River, Peru, is sister to the Yasuní + Juyuintza in Ecuador. The Juyuintza samples are sister to each other. All genetic distances reported below are uncorrected distances for the gene *12S*. Genetic distances between Ere River, Peru, and the Ecuadorian populations (Juyuintza and Yasuní) range from 5.3 to 5.9%; distances between Yasuní and Juyuintza are both 1.2%. Samples from Juyuintza are identical to each other. The genetic distances between Ere River and the other populations are above distances between several closely related species in *Tepuihyla* and *Osteocephalus* (e.g., *Osteocephalus buckleyi*-*Osteocephalus cabrerai* 2.8%, *Osteocephalus oophagus*-*Osteocephalus taurinus* 1.3–1.8%, *Osteocephalus cannatellai*-*Osteocephalus cabrerai* 2.6%, *Osteocephalus cannatellai*-*Osteocephalus vilmae* 2.4%, *Tepuihyla rodriguezi*-*Tepuihyla exophthalma* 4.5–4.6%, *Tepuihyla rodriguezi*-*Tepuihyla edelcae* 1.3–1.5%).

**Figure 3.**
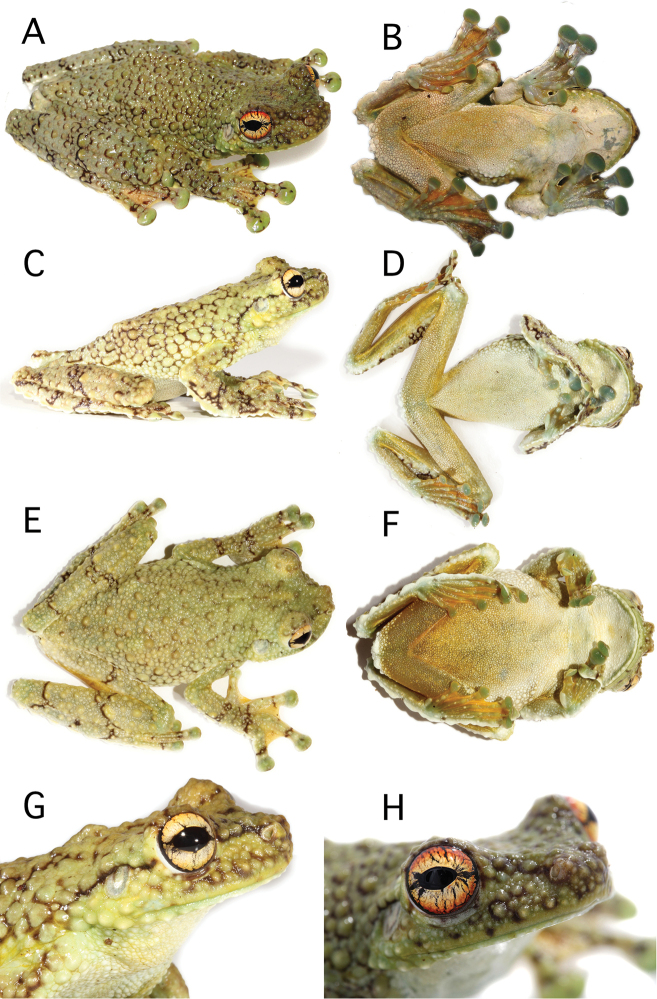
External morphology of *Tepuihyla shushupe* sp. n. and *Tepuihyla tuberculosa* comb. n. **A–B** *Tepuihyla shushupe* sp. n. CORBIDI 12513 (holotype), adult male, SVL = 85.3 mm, Ere river, Peru **C–D** *Tepuihyla tuberculosa* comb. n. QCAZ 55423, adult male, SVL = 83.1 mm, Parque Nacional Yasuní, Tambococha, Ecuador; **E–F** *Tepuihyla tuberculosa* comb. n., QCAZ 55413 juvenile, SVL = 58.1 mm, Parque Nacional Yasuní, Tambococha, Ecuador **G–H** iris coloration of *Tepuihyla tuberculosa* comb. n. (QCAZ 55423) and *Tepuihyla shushupe* sp. n., (CORBIDI 12513). Photographs: **A–B, H** by P. Venegas, **C–G** by D. Quirola.

**Figure 4.**
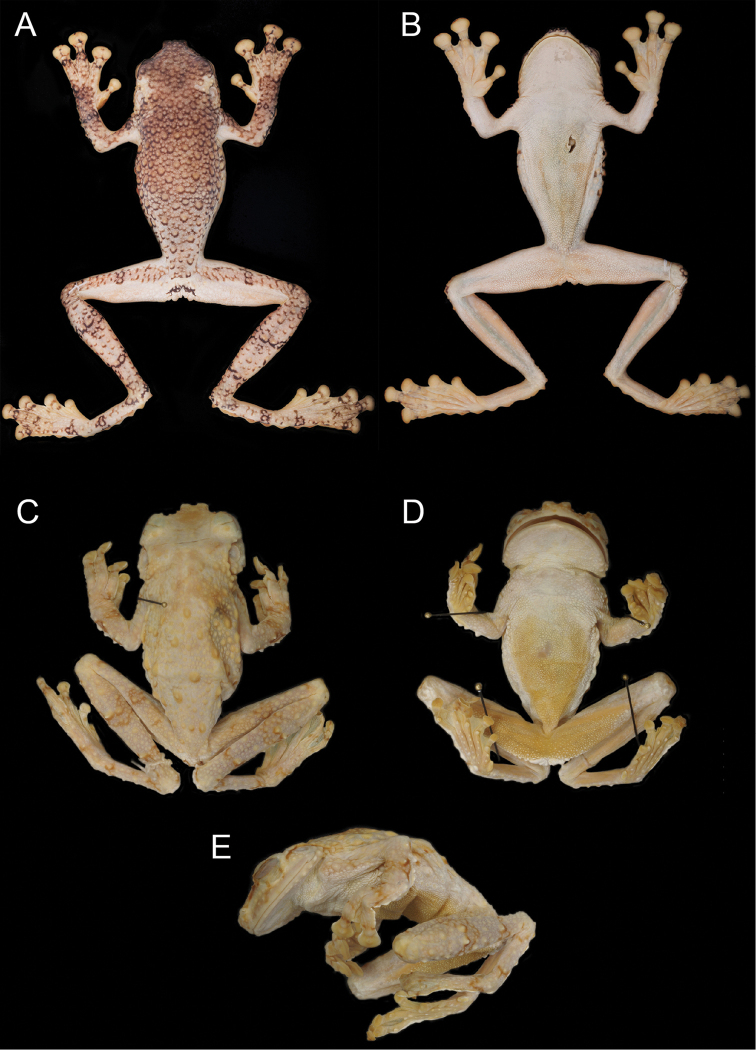
Holotypes of *Tepuihyla shushupe* sp. n. (**A–B** CORBIDI 12513) and *Tepuihyla tuberculosa* comb. n. (**C–E** BMNH 1947.2.13.34). Photos of *Tepuihyla tuberculosa* comb. n. by M. R. Bustamante.

Morphologically, the individual from Ere River differs from all other populations (in parenthesis) in having a cream iris with red periphery (entirely cream to reddish cream; Fig. 3G vs. 3H) and dorsum with small tubercles intermixed with abundant large tubercles (small tubercles intermixed with few large tubercles; Fig. 4A vs. 4C and 5). We found differences in advertisement calls between Río Ere and Juyuintza. Calls consist of a cackle of short notes repeated at a fast rate (Table 3; Fig. 6). Note amplitude and note rate increases markedly along the first half of the call. The call from Juyuintza differs from the call from Ere River in temporal and spectral variables. The former has shorter duration, fewer notes, and lower note rate (Ere River 3.37–3.38, 3.37 ± 0.01; Juyuintza 2.63–2.86, 2.74 ± 0.16; Table 3). Harmonic structure is markedly different between calls. The call from Ere River has two harmonics with similar energy content while the call from Juyuintza has one well-defined harmonic with high energy and a second harmonic with low energy, barely evident in the power spectra (Fig. 6).

**Figure 5.**
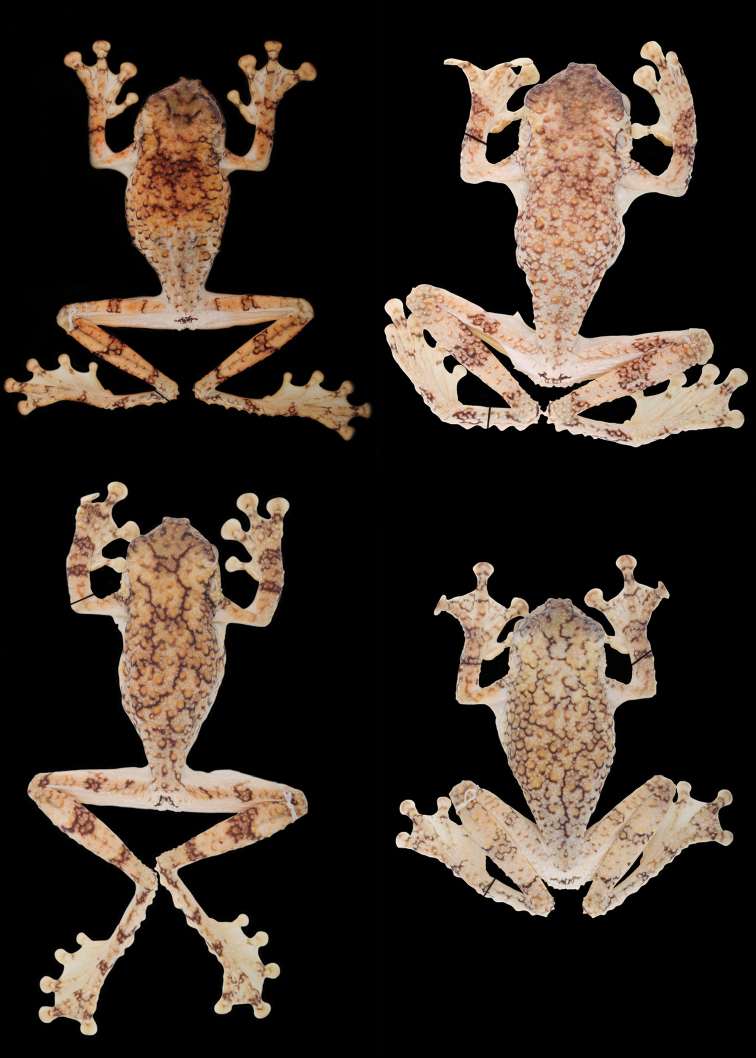
Variation in dorsal coloration of preserved specimens of adult *Tepuihyla tuberculosa* comb. n. Left to right, upper row: QCAZ 52855 (male), QCAZ 53542 (male); lower row: QCAZ 53147 (male), QCAZ 55423 (male). See Suppl. material 2 for locality data. Specimens are shown at the same scale.

**Figure 6.**
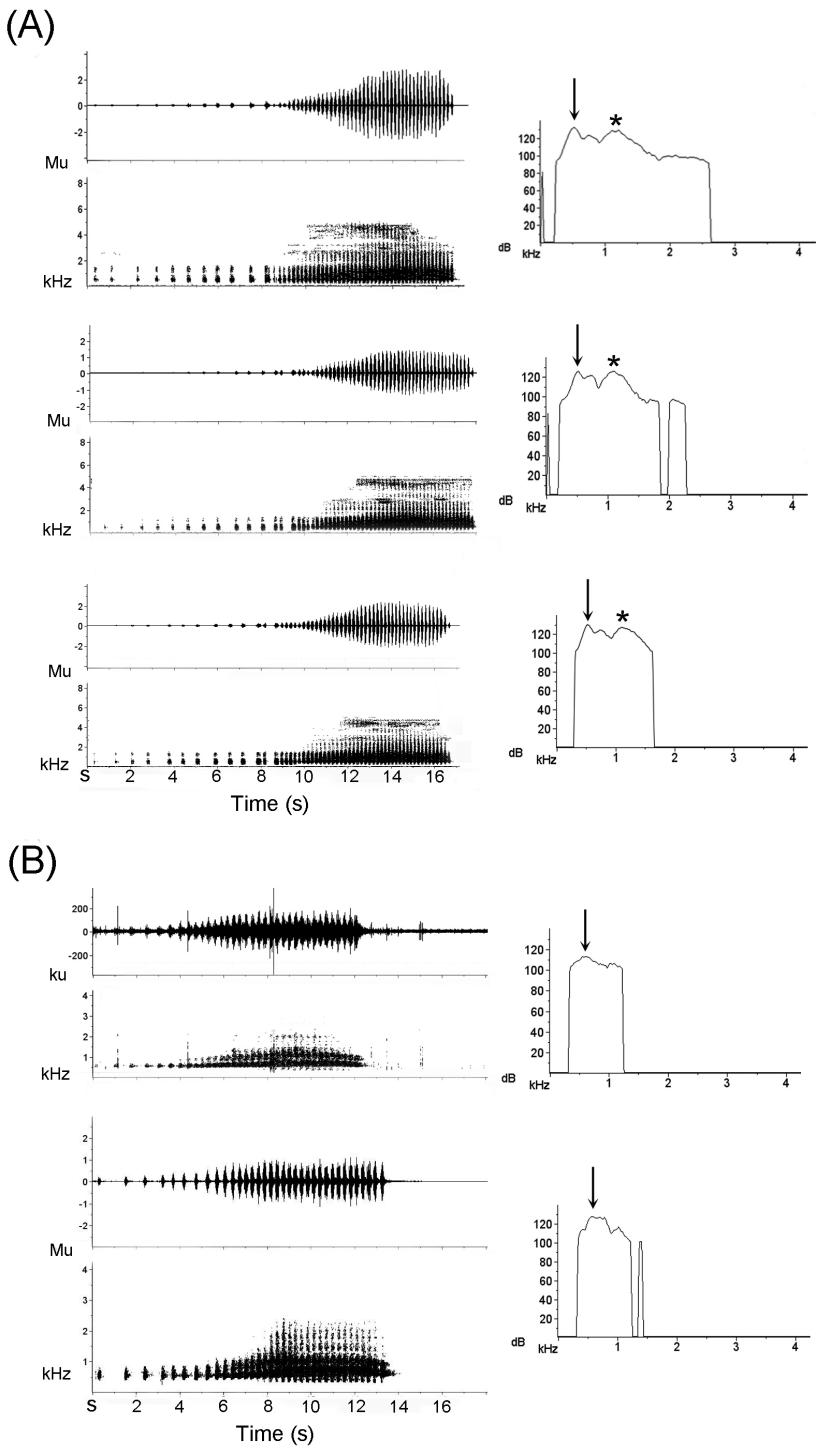
Oscillogram, sound spectrogram (first column), and power spectrum (second column) of advertisement calls of *Tepuihyla shushupe* sp. n. (**A**) and *Tepuihyla tuberculosa* comb. n. (**B**). Note differences in call length and number of notes (first column). Both species have the dominant frequency (arrows) in the first harmonic but in *Tepuihyla shushupe* sp. n. the second harmonic has similar energy content (asterisk), while in *Tepuihyla tuberculosa* comb. n. it has much less.

**Table 3.** Quantitative and qualitative characteristics of the advertisement call of *Tepuihyla* from Ecuador and Peru. The recording in Peru was made in the Loreto Department, Putumayo River basin, Rio Ere on 18 October 2012, air temperature of 21.3 C. The recording in Ecuador was made in Pastaza Province, Juyuintza on 22 June 2012, air temperature 23.9 C.

	*Tepuihyla shushupe* sp. n. (CORBIDI 12513)	*Tepuihyla tuberculosa* comb. n. (QCAZ 53699)
Calls analyzed (specimens)	3(1)	2 (1)
Call duration (s)	16.4–17.2 (16.7±0.47)	12.2–13.1
Call rate (calls/minute)	0.45	0.37
Call interval (s)	117.024	~150
Call rise time (s)	12.7–15.7 (13.74±1.69)	6.96–11.8
Notes per call (total notes analyzed)	56–59 (171)	35 (70)
Note rate (average ± sd)	3.37–3.38 (3.37±0.01)	2.63–2.86 (2.74±0.16)
Note length (average ± sd)	0.08–0.26 (0.13±0.04)	0.17–0.43 (0.24±0.05)
Note rise time (average ± sd)	0.02–0.14 (0.06±0.03)	0.05–0.23 (0.1±0.03)
Note shape	0.2–0.94 (0.44±0.13)	0.19–0.98 (0.43±0.13)
Frequency band	96.9–5102.9	150.7–2452.1
Fundamental frequency	404.3–585.9 Hz (500.08±28.3)	515.6–796.9 Hz (619.1±77.2)
Dominant frequency (DF)	515.6 Hz (1st harmonic)	562.5–632.8 (1st harmonic)
DF beginning (% of notes)	404.3–585.9 Hz (100% in the 1st harmonic)	515.6–703.1 Hz (100% in the 1st harmonic)
DF middle (% of notes)	509.8–1210 Hz (75% in the 2nd harmonic)	585.9–1195.3 Hz (90% in the 1st harmonic)
DF end (% of notes)	445.3–539.1 Hz (100% in the 1st harmonic)	585.9–679.7 Hz (100% in the 1st harmonic)
Fundamental frequency ratio	0.75+0.44	0.97+0.06
Frequency modulation	-1.0–0.0	0.0–5.3

The combined evidence from genetics, morphology, and bioacoustics indicates the existence of two species within “*Ecnomiohyla tuberculosa*”. Specimens from Yasuní and Juyuintza have similar coloration and dorsal tuberculation to the holotype of *Ecnomiohyla tuberculosa* (BMNH 1947.2.13.34), therefore we assign them to *Ecnomiohyla tuberculosa* *sensu stricto*. The specimen from Rio Ere belongs to an undescribed species that we describe in the following sections. Our species description is based on a single specimen and therefore we cannot characterize the variation in the new species. However, we examined 11 specimens of *Ecnomiohyla tuberculosa*, which allow us to characterize *Ecnomiohyla tuberculosa* morphological variation and show that the single specimen of the new species falls outside the range of variation of *Ecnomiohyla tuberculosa*. Hence, we demonstrate that the new species is, in fact, distinct from *Ecnomiohyla tuberculosa*. Our morphological evidence is corroborated by genetic and bioacoustic characters.

### Taxonomic Review of *Ecnomiohyla tuberculosa*

Recent phylogenies of Hylidae (Wiens et al. 2010) and all amphibians (Pyron 2014) show, with strong support, that *Ecnomiohyla* is part of Hylini, a tribe composed by the genera *Acris*, *Anotheca*, *Bromeliohyla*, *Charadrahyla*, *Diaglena*, *Duellmanohyla*, *Ecnomiohyla*, *Exerodonta*, *Hyla*, *Isthmohyla*, *Megastomatohyla*, *Plectrohyla*, *Pseudacris*, *Ptychohyla*, *Smilisca*, *Tlalocohyla*, *Triprion*. Wiens et al. (2010) and Pyron (2014) phylogenies only included 3 out of 14 species of *Ecnomiohyla*: *Ecnomiohyla milaria*, *Ecnomiohyla minera*, and *Ecnomiohyla miotympanum*. Batista et al. (2014) analyzed one fragment of the mitochondrial gene 16S from seven species of *Ecnomiohyla*. Their outgroup was limited to two genera within Hylini and was not suitable to assess the phylogenetic position of *Ecnomiohyla* within Hylidae. Nevertheless, they provided the most complete phylogeny for *Ecnomiohyla* to date.

In our phylogeny, *Ecnomiohyla tuberculosa* is part of the tribe Lophiohylini. Because all other species of *Ecnomiohyla* are part of the tribe Hylini (Pyron 2014; Wiens et al. 2010; Suppl. material 2), the phylogenetic position of *Ecnomiohyla tuberculosa* renders the genus *Ecnomiohyla* polyphyletic and the genus *Tepuihyla* paraphyletic (Fig. 1). We solve both problems by assigning *Hyla tuberculosa* Boulenger 1882 to the genus *Tepuihyla*. This change results in *Tepuihyla tuberculosa* (Boulenger 1882) comb. n.

Below we present the species account for *Tepuihyla tuberculosa* and we describe the new species from Ere River, Peru. Under this new taxonomy, the genus *Tepuihyla* contains ten species distributed in the Pantepui region of Venezuela, Guyana and in the Amazon region of Brazil, Colombia, Ecuador, and Peru (AmphibiaWeb 2016; Salerno et al. 2015; Kok et al. 2015).

#### Tepuihyla tuberculosa

Taxon classificationAnimaliaAnuraHylidae

(Boulenger, 1882)

Hyla tuberculosa Boulenger 1882. Holotype BMNH 1947.2.13.34
Ecnomiohyla tuberculosa : Faivovich et al. 2005

##### Holotype.

Sub adult female with SVL 67.6 mm (Fig. 4C–E). The description of the holotype provided by Boulenger (1882) is adequate.

##### Diagnosis.

**Figure 7.**
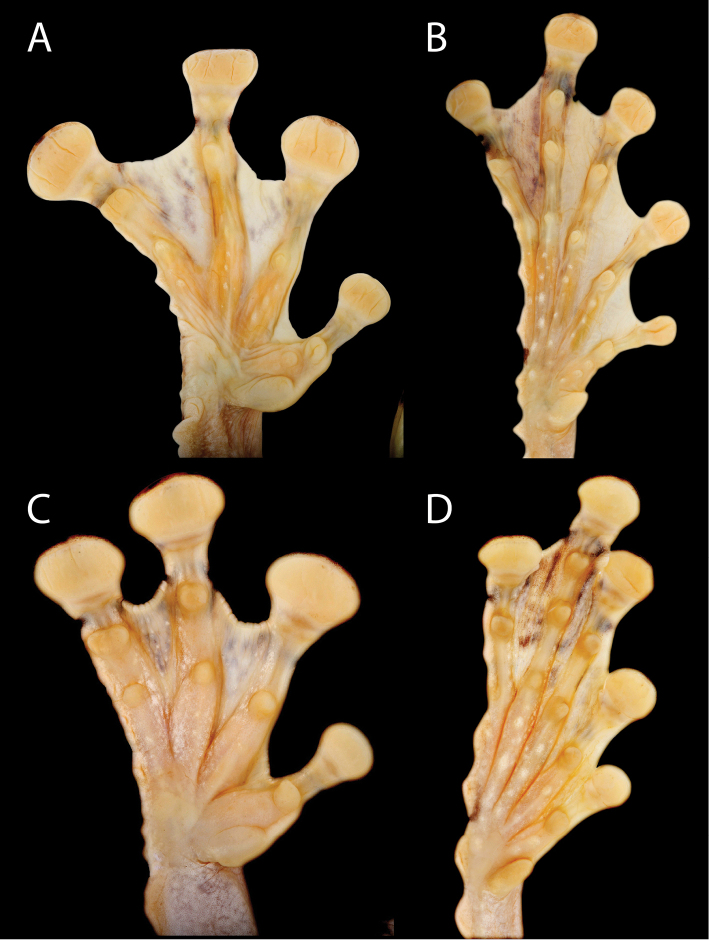
Ventral views of the left hand and foot of *Tepuihyla*. **A–B** *Tepuihyla tuberculosa* comb. n. (Parque Nacional Yasuní, Ecuador, SVL = 86.2 mm, QCAZ 52855, adult male) **C–D** *Tepuihyla shushupe* sp. n. (Ere River, Peru, SVL = 85.4 mm, CORBIDI 12513, adult male).

In this section coloration pertains to preserved specimens unless otherwise noted. A large-sized *Tepuihyla* differing from other known species in the genus by the following combination of characters: (1) SVL in males 79.3–86.2 mm (*n* = 5), SVL in females 67.6–85.7 mm (*n* = 2); (2) skin on dorsum coarsely tuberculate, covered by small tubercles with scattered large tubercles; tubercles without keratinized tips; (3) skin on flanks covered by large tubercles; (4) webbing between fingers extensive but without reaching the proximal border of the disks, hand webbing formula **I**2 —2 **II**1 —2 **III**1½—1**IV to I**2—2+**II**1—2**III**2 —1**IV** (Fig. 7A); webbing between toes extensive and reaching the proximal border of the disks on at least three toes, foot webbing formula **I**0+—1+**II**0+—1 **III**0+—1+**IV**1+— 0+**V** to **I**1+—1½**II**1—1+**III**1+—1½**IV**1½— 0+**V** (Fig. 7B); (5) dorsal coloration in life greenish cream or brownish cream with scattered dark brown reticulations, anterior and posterior surfaces of thighs and hidden surfaces of shanks yellowish orange; (6) ventral coloration whitish cream and webbing between fingers and toes pale orange; (7) suborbital mark absent, clear labial stripe present, faint and with some brown spots; (8) coloration on flanks similar to dorsal coloration except for greenish yellow axilla and groins and whitish gray ventrolateral area; (9) skin on upper surface of head not co-ossified with underlying cranial elements, cranial crest slightly exostosed; (10) in life, bones green; (11) small triangular dermal flaps form serrate fringe along the ventrolateral margin of the forearm and along the outer edge of Finger IV; heels bearing two or three enlarged fleshy conical tubercles surrounded by few smaller round tubercles; small triangular dermal flaps form serrate fringe along ventrolateral margin of tarsus and outer margin of Toe V; (12) adults, in life, have cream iris with a coppery hue and scattered thin black reticulations; (13) vocal sac is white, single and subgular, (14) a juvenile is similar to adults in coloration but with dorsal background coloration brownish cream and a black medial stripe in the iris; (15) larvae unknown.

*Tepuihyla tuberculosa* differs from all congeneric species (in parenthesis), except *Tepuihyla shushupe* sp. n., in having a larger size: SVL in males 79.3–86.2 mm (*n* = 5), SVL in females 67.6–85.7 mm (*n* = 2) (maximum SVL 59.2 mm in other *Tepuihyla*), having extensive webbing in the hands (basal webbing) and a serrate fringe along the ventrolateral margin of the forearm (absent). Other differences are listed on Table 4.

**Table 4.** Qualitative morphological characters of frogs of the genus *Tepuihyla*. Data was obtained from Ayarzagüena et al. 1992; Duellman and Hoogmoed 1992; Gorzula and Señaris 1998; Kok and Kalamandeen 2008; Kok et al. 2015; Mijares-Urrutia et al. 1999).

	Serrate fringes on limbs	Maximum size females (mm)	Dorsum	Tubercles on jaw	Webbing between fingers	Vocal sac
*Tepuihyla aecii*	absent	36.8	smooth (females) to spiculate (males)	absent	absent	subgular
*Tepuihyla edelcae*	absent	45.7	smooth (females) to spiculate (males)	absent	absent	subgular
*Tepuihyla exophthalma*	absent	42.5	smooth with few tubercles	absent	absent	subgular
*Tepuihyla luteolabris*	absent	59.2	granular	absent	absent	subgular
*Tepuihyla rodriguezi*	absent	50.3	smooth (females) to spiculate (males)	absent	absent	subgular
*Tepuihyla shushupe* sp. n	forelimbs and hindlimbs	--	tuberculate	present	extensive	subgular
*Tepuihyla tuberculosa* comb. n.	forelimbs and hindlimbs	85.7	tuberculate	present	extensive	subgular
*Tepuihyla obscura*	absent	38.4	smooth (females) to spiculate (males)	absent	absent	subgular
*Tepuihyla warreni*	absent	36.2	smooth	absent	basal	subgular

**Figure 8.**
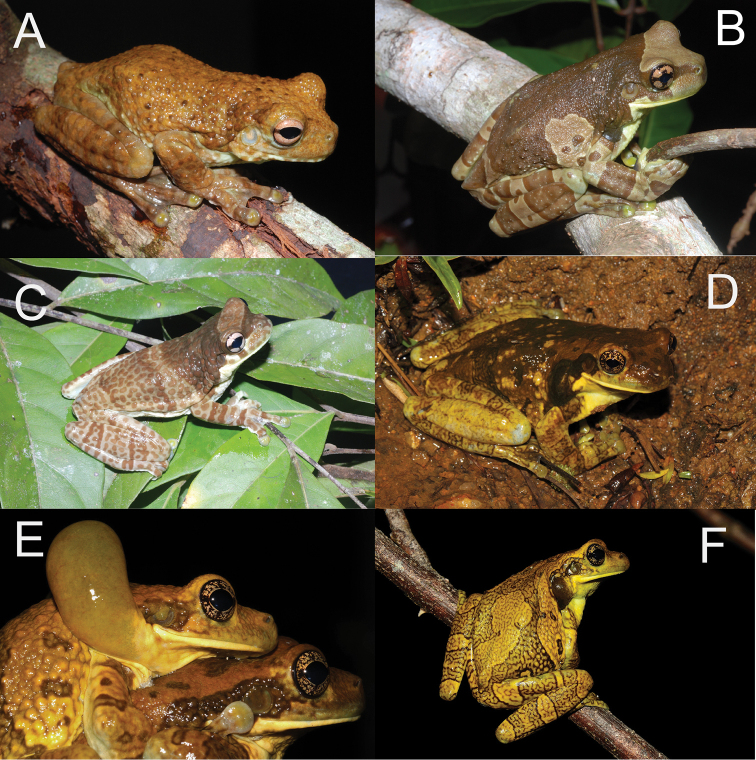
Dorsolateral views of Amazonian *Trachycephalus*. **A** *Trachycephalus helioi*, Juruti, Pará, Brazil **B** *Trachycephalus resinifictrix*, Juruti, Pará, Brazil **C** *Trachycephalus cunauaru*, Santa Izabel do Rio Negro, Amazonas, Brazil **D–E** *Trachycephalus typhonius*, Village de Ouanary, French Guiana **F** *Trachycephalus typhonius*, Matiti, French Guiana. Photographs: **A–C** by M. Gordo, **D–E** by Daniel Baudain, **F** by Vincent Premel.

*Tepuihyla tuberculosa* is most similar to *Tepuihyla shushupe* sp. n. It differs from *Tepuihyla shushupe* sp. n. (character states in parenthesis) in having cream iris in life (iris cream with reddish periphery), dorsum covered with small tubercles intermixed with few large tubercles (dorsum covered with small tubercles intermixed with abundant large tubercles; Figs 3–5); in preservative, its dorsum is light cream or cream with a coppery hue and brownish cream to creamy coppery large tubercles (dorsum light brown with large brownish cream or creamy coppery tubercles with a dark brown posterior border). *Tepuihyla tuberculosa* also resembles the Amazonian *Trachycephalus cunauaru* and *Trachycephalus resinifictrix* but differs from both species in having serrate fringes on the limbs and tubercles on the lower jaw (both absent in *Trachycephalus*; Fig. 8). *Tepuihyla tuberculosa* is easily distinguished from all other large Amazonian treefrogs in having fully webbed hands and feet, coarsely tubercular skin on dorsum, and serrate dermal fringes on the outer margin of the forearm and foot. *Cruziohyla craspedopus* and *Dendropsophus marmoratus* also have dermal fringes on the outer margin of the foot, but the former is distinguished by its deep green dorsum, vertically elliptical pupils in a bicolored iris (pale silver with yellow borders), smooth skin on dorsum and elongate calcar on the heel. The smaller *Dendropsophus marmoratus* exhibits a bright yellow belly with black spots or mottling (uniform yellowish-tan in *Tepuihyla tuberculosa*).

##### Variation.

In this section, coloration refers to preserved specimens unless otherwise noted. Morphometric data for adult specimens are summarized in Table 5, whereas variation in dorsal coloration of preserved specimens is shown in Figure 5. Dorsal coloration varies between light cream (e.g., QCAZ 55423) and cream suffused with a coppery hue (e.g., QCAZ 52855) with brownish cream or creamy coppery enlarged tubercles, bearing scattered dark brown reticulations or marks. In all specimens the belly and ventral areas of thighs are lighter than those of the holotype probably because they were collected more recently. Head is rounded in dorsal view, wider than long; the snout is truncate in dorsal view and truncate (e.g. QCAZ 32716) in profile. The largest male has 86.2 mm SVL (average = 82.9 ± 2.7 mm, *n* = 5) and the largest female 85.7 mm (Table 5).

**Table 5.** Descriptive statistics for measurements of adult Amazonian *Tepuihyla*. Mean ± SD is given with range in parenthesis. Abbreviations are: SVL = snout-vent length; FOOT = foot length; HL = head length; HW = head width; ED = eye diameter; TD = tympanum diameter; TL = tibia length; FL = femur length. All measurements are in mm.

	*Tepuihyla shushupe* sp. n. Male CORBIDI 12513	*Tepuihyla tuberculosa* comb. n. Female QCAZ 32716	*Tepuihyla tuberculosa* comb. n. males (n = 5)
SVL	85.4	85.7	82.9 ± 2.9 (79.3–86.2)
FOOT	35.5	35.8	35.53 ± 2.4 (33.5–39.5)
HL	26.6	25.7	26.0 ± 1.0 (25.1–27.5)
HW	28.9	29.2	28.9 ± 1.6 (27.8–31.7)
ED	7.7	7.5	7.5 ± 0.9 (6.3–8.9)
TD	5.5	4.8	5.3 ± 0.5 (4.9–5.8)
TL	46.9	45.6	46.3 ± 2.2 (44.1–49.9)
FL	35.9	36.6	36.8 ± 1.3 (35.0–38.3)

##### Coloration in life.

Dorsal coloration is greenish cream with scattered brown marks or reticulations, top and sides of head are more greenish in some specimens (e.g. QCAZ 52855); lips greenish with scattered small dark brown marks; supratympanic fold delineated by a thin brown edge, tympanic membrane lighter than the background; flanks with coloration similar to dorsum; axilla and groin pale orange, ventrolateral region whitish cream, limbs with faint brown transversal bands; dorsal surfaces of hands and feet, including webbing, greenish cream with scattered dark brown marks; discs pale green. Throat, chest and belly are white but some specimens have an orange hue on the belly (e.g. QCAZ 55423); serrate fringes along forearms and tarsus white; webbing, thighs and concealed surfaces of shanks orange. Iris is cream with a light coppery hue bearing some scattered black venations. Juvenile specimen QCAZ 55413 is similar to adult specimens but has a brownish cream dorsum with scattered faint brown blotches (Fig. 3E–F).

##### Advertisement call.

Quantitative characteristics of the advertisement call of *Tepuihyla tuberculosa* (QCAZ 53699) are detailed in Table 3. The call consists of a cackle of short notes repeated at a fast rate with amplitude modulation (Fig. 6). Note amplitude and note rate increase markedly along the first half of the call, decreasing at the end. The call has two harmonics but most of the energy located on the first. Fundamental frequency of the notes ranges from 515.6 to 796.9 Hz (mean = 619.1, SD = 77.2). Two notes located at ~50% of the call duration have their greatest energy in the second harmonic, at 1078.1 and 1195.3 Hz. All other notes have their greatest energy in the first harmonic. The dominant frequency of the entire call ranges from 562.5 to 632.8 Hz. Interestingly, the call of this species is feared by natives in the lower Pastaza basin (Shiwiar, Sapara, Shuar, Achuar people), because it is commonly confused with the “calling” of the bushmaster *Lachesis muta* (Squamata: Viperidae). This belief almost certainly incorrect as *Lachesis muta* cannot vocalize. See also the Etymology section in *Ecnomiohyla shushupe* sp. n. description.

##### Distribution and natural history.

**Figure 9.**
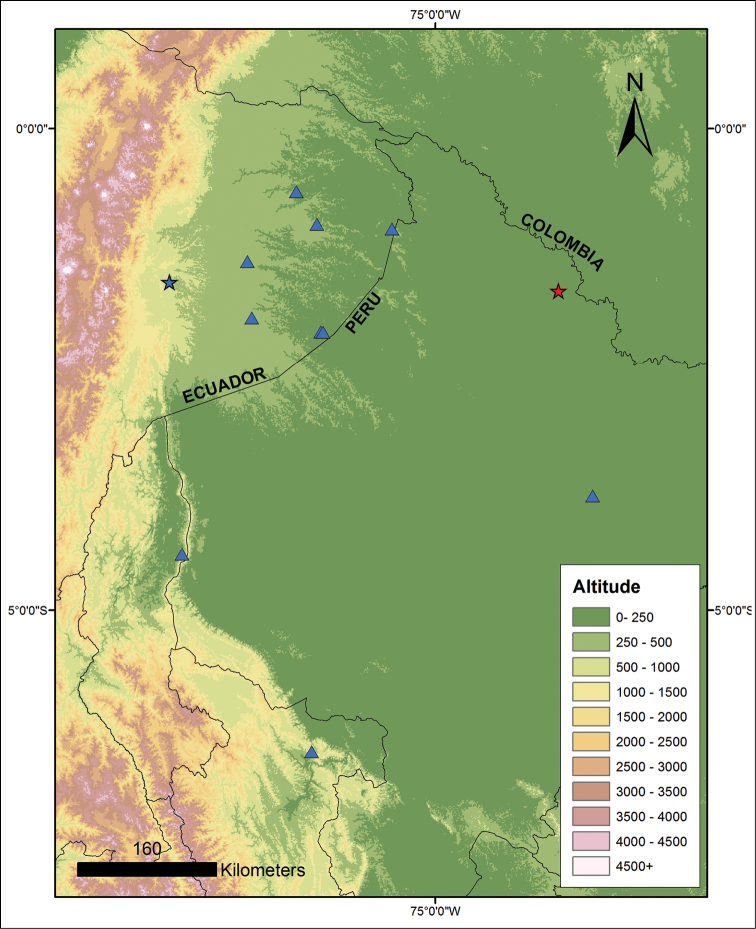
Records of *Tepuihyla shushupe* sp. n. and *Tepuihyla tuberculosa* comb. n. *Tepuihyla shushupe* sp. n., red star; *Tepuihyla tuberculosa* comb. n., blue triangles and blue star for type locality. Locality data from specimens deposited at Natural History Museum (BMNH), London, Centro de Ornitología y Biodiversidad (CORBIDI), and Museo de Zoología, Pontificia Universidad Católica del Ecuador (QCAZ).

Localities documented for this species are shown in Figure 9. Duellman (1974) reported four specimens: two from Pastaza province in Ecuador, one from the mouth of Rio Santiago in northern Peru, and one juvenile from Rio Uaupes at junction of Rio Querari, Amazonas, Brazil. Given its geographic location, the latter probably corresponds to *Tepuihyla shushupe* sp. n., but its identity requires confirmation. We recorded five additional localities in the Amazon lowlands of Ecuador and two in Peru (Fig. 9). Elevation range is 132 to 1076 m above sea level. The southernmost and highest locality is Cordillera Escalera, San Martin department, in northeastern Peru (CRBIIAP 1252).

**Figure 10.**
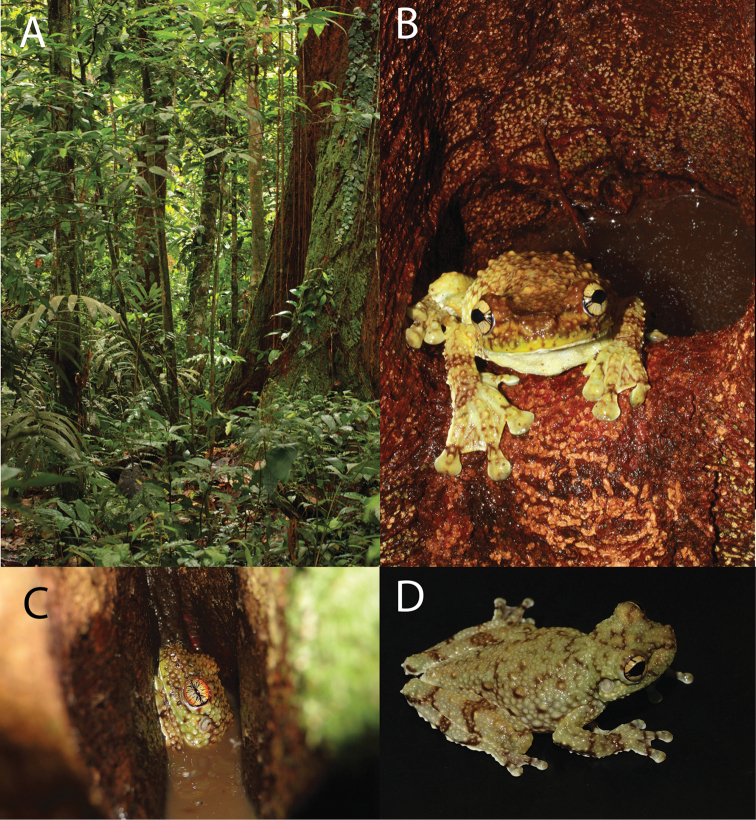
Life individuals and habitat of *Tepuihyla shushupe* sp. n. and *Tepuihyla tuberculosa* comb. n. **A** habitat of *Tepuihyla tuberculosa* comb. n. at Juyuintza, Provincia Pastaza, Ecuador **B** Adult male of *Tepuihyla tuberculosa* comb. n. (QCAZ 53542) sitting at the entrance of the tree hole where he was calling **C** Adult male of *Tepuihyla shushupe* sp. n. (CORBIDI 12513, holotype) partly submerged in the water accumulated on the tree hole where he was calling **D** *Tepuihyla tuberculosa* comb. n. (CRBIIAP 1839) from the vicinity of Puerto Almendras, Loreto, Peru. Photographs: **A, B** by M. Ortega, **C** by A. Del Campo, **D** by G. Gagliardi-Urrutia.

All specimens with ecological data were collected at night perching on vegetation 1.5 to 3.0 m above the ground. One specimen from Yasuní (QCAZ 52855) and one from Juyuintza (QCAZ 53542; Fig. 10B) were collected on primary forest (M. Read and H. M. Ortega-Andrade field notes). The specimen recorded at Juyuintza was calling from a tree hole 1.5 m above the ground in *Terra Firme* forest. The hole had a depth of ~15 cm and a diameter of 10 cm, and was ¾ flooded with water. Two individuals were calling nearby, at distances of approximately 100 to 200 m. No amplectant pairs, clutches, or tadpoles have been observed.

##### Conservation status.

The scarcity of records of *Ecnomiohyla tuberculosa*, even at thoroughly sampled localities, like Yasuni National Park, could be a consequence of small populations sizes or an artifact of low capture probabilities. Our finding of a male calling from a tree hole containing water suggests that *Ecnomiohyla tuberculosa* breeds on tree holes and is unlikely to be found on ground-level surveys. Therefore, lack of records probably results from low capture probabilities consequence of microhabitat preferences. Because population status is unknown, we suggest that the Red List category of *Tepuihyla tuberculosa* is Data Deficient according to IUCN (2001) guidelines. Searches of the species will benefit from audio sampling to detect the advertisement call described here.

#### Tepuihyla shushupe sp. n.

Taxon classificationAnimaliaAnuraHylidae

http://zoobank.org/8045B112-6377-491C-ACFD-3DF3BA2192F7

Ecnomiohyla tuberculosa : Venegas and Gagliardi-Urrutia (2013). Species misidentification in their Figures 8B and 8G.

##### Holotype.

(Figs 3–4, 6, 10C) CORBIDI 12513, adult male from Peru, Loreto department, Maynas province, head waters of rivers Ere and Campuya, Putumayo River basin (1.6790°S, 73.7197°W), 145 m above sea level, collected by P. J. Venegas and G. Gagliardi-Urrutia on 19 October 2012.

##### Diagnosis.

In this section coloration pertains to preserved specimens unless otherwise noted. A large-sized *Tepuihyla* differing from other species in the genus by the following combination of characters: (1) SVL in males 85.4 mm (n = 1), females unknown; (2) skin on dorsum coarsely tuberculate, covered by small tubercles intermixed with large tubercles; tubercles without keratinized tips; (3) skin on flanks similar to skin on dorsum; (4) webbing between fingers extensive but without reaching the proximal border of disks; hand webbing formula **I**2—2**II**1— 2**III**1½—1+**IV** (Fig. 7C); webbing between toes extensive, reaching the proximal border of the disks on at least three toes; foot webbing formula **I**1—1+**II**0+—1+**III**0+—1½**IV**1½— 0+**V** (Fig. 7D); (5) dorsal coloration in life brownish green, anterior and posterior surfaces of thighs and hidden surfaces of shanks yellowish orange; (6) ventral coloration whitish gray and webbing between fingers and toes pale orange; (7) suborbital mark absent, clear labial stripe present, faint and with some brown spots; (8) coloration on flanks similar to dorsal coloration except for greenish yellow axilla and groins and whitish gray ventrolateral area; (9) skin on upper surface of head not co-ossified with underlying cranial element, cranial crest slightly exostosed; (10) in life, bones green; (11) small triangular dermal flaps form serrate fringe along the ventrolateral margin of the forearm and along the outer edge of Finger IV; heels bearing two enlarged fleshy conical tubercles surrounded by few smaller round tubercles; small triangular dermal flaps form serrate fringe along ventrolateral margin of tarsus and outer margin of Toe V; (12) in life, iris reddish peripherally turning cream towards to the center, with irregular black reticulations; (13) vocal sac is white, single and subgular, (14) juveniles unknown; (15) larvae unknown.

*Tepuihyla shushupe* differs from all species of the genus (in parenthesis), except *Tepuihyla tuberculosa*, in being larger, having extensive hand webbing (basal webbing) and a serrate fringe along the ventrolateral margin of the forearm (absent). Other differences are listed on Table 4.

*Tepuihyla shushupe* is most similar to *Tepuihyla tuberculosa*. It differs from *Tepuihyla tuberculosa* (character states in parenthesis) in having, in life, a cream iris with red periphery (iris cream without red periphery), dorsum covered by small tubercles intermixed with abundant large tubercles (dorsum covered by small tubercles intermixed with few large tubercles); in preservative, dorsum cream with a brownish mantle (dorsum light cream or cream suffused with a coppery hue) and the posterior border of large dorsal tubercles dark brown (tubercles brownish cream or creamy coppery). In coloration, size, and texture of dorsal skin *Tepuihyla shushupe* resembles the Amazonian *Trachycephalus resinifictrix* and *Trachycephalus cunauaru*. It can be distinguished from both species by the presence of serrate fringes on the limbs and tubercles in the lower jaw (both absent in *Trachycephalus*) and the iris having red periphery and lacking a vertical black bar below the pupil (iris without red and with a vertical back bar below the pupil in both *Trachycephalus*).

##### Description of the holotype.

Adult male (CORBIDI 12513), 85.4 mm SVL, head length 26.6, head width 28.9, eye diameter 7.7, tympanum diameter 5.5, femur length 35.9, tibia length 46.9, foot length 35.9. Head rounded in dorsal view, wider than long (HL/HW = 92%); snout truncate in dorsal view and slightly protruding in profile; nostrils are directed laterally, near tip of the snout; top of head tuberculate; canthus rostralis concave in dorsal view, straight in profile; skin on upper surface of head not co-ossified with underlying cranial elements, cranial crests slightly exostosed; loreal region concave; skin on dorsal surface of head, body and limbs coarsely tuberculate, tubercles are either small or large and lack keratinous spines; lower eyelid pigmented; distinct supratympanic fold formed by a row of round tubercles running from midpoint of posterior margin of eye; tympanum prominent, opaque, smooth, 71% of eye diameter, separated from eye by 3.8 mm; small triangular dermal flaps form serrate fringe along the ventrolateral margin of the forearm and the outer edge of Finger IV, fringe more conspicuous on forearm that on finger; hands moderate in length (HAL/SVL = 31.2%); Finger lengths I < II < IV < III, terminal disk on Finger I 63.2% of diameter of disks on Fingers II–IV; diameter of Fingers II-IV ~1.2 times tympanum diameter; distal subarticular tubercles on Fingers I–III large and rounded, on Finger IV larger and bifid; supernumerary tubercles indistinct; prepollical tubercle large, obtuse and elliptical; bony prepollical projection absent; keratinous nuptial pad with dark epidermal projections covering the surface of the thumb up to the distal edge of its subarticular tubercle; fingers considerably webbed although web does not reach base of disks; webbing formula **I**2—2**II**1— 2**III**1½—1¼**IV**; legs relatively long and slender (tibia length 54.8% of SVL), thigh length 35.9 mm; heels bearing two enlarged conical tubercles surrounded by few lower round tubercles; fleshy, dermal flaps form serrate fringe along ventrolateral margin of tarsus and outer margin of Toe V extending to base of disk, scallops are deeply incised, largest on tarsus, smaller along toe; outer metatarsal tubercle small, inner metatarsal tubercle moderately large, ovoid, and flat; toe lengths I < II < III = V < IV; disks on toes ~70% of diameter of disks on fingers, equal between Toes III–V; subarticular tubercles round; supernumerary tubercles present, ill defined; webbing in toes extensive, reaching the base of disks on at least three toes; webbing formula **I**1—1**II**1—1¼**III**¾—1**IV**1— ¾**V**; gular skin smooth with small pointed tubercles along the jaw , venter finely granulate, weak granulation on undersides of arms and finely on ventral surfaces of thighs, smooth skin on anterior surfaces of thighs and ventral surfaces of legs; cloacal opening directed posteriorly at upper-level of thighs, some indistinct small tubercles on the upper edge of vent, skin under the vent covered by small flattened but conspicuous tubercles with two enlarged prominent fleshy tubercles posterolateral to the vent (one on each side); tongue cordiform; vomerine ridges transverse, oblique in the middle, narrowly separated medially, placed between the posterior margins of the large subrectangular choanae; vomerine teeth 6 to 11; vocal slits present.

##### Color of holotype in life

**(Figs 3, 10).** Based on digital photographs. Dorsal and lateral surfaces of body and limbs pale green; posterior edge of each enlarged tubercle, dark brown; lips paler than the rest of head; lower flanks, forearms, tibia, and tarsus white; dorsal surfaces of hindlimbs with narrow dark brown transversal stripes; axillar region, groins, anterior and posterior surfaces of thighs, and hidden surfaces of shanks greenish yellow; dorsally, webbing between fingers and toes pale greenish yellow with scattered dark brown marks, ventrally greenish orange. Venter whitish gray with an orange tone on belly and ventral surfaces of thighs; ventral surfaces of arms and elbows greenish yellow; ventral surfaces of tibia, tarsus, palms, fingers, soles, toes and discs water green; limb bones green; iris cream with red periphery and irregular black reticulations.

##### Color of holotype in preservative

**(Figs 4A–B, 5C–D).** Dorsal surfaces of head, body and forelimbs, including fingers, are tan; large dorsal tubercles have a dark brown margin; dorsal surfaces of hindlimbs and toes dirty cream bearing scattered light tubercles with dark brown margins and narrow dark brown transversal stripes; posterior surfaces of thighs light cream; dorsal surfaces of discs on fingers and toes are light cream with dark brown marks. Venter is whitish cream.

##### Advertisement call.

Quantitative characteristics of the advertisement call *Tepuihyla shushupe* (CORBIDI 12513) are detailed in Table 3. The call consists of a cackle of short notes repeated at a fast rate with amplitude modulation (Fig. 6). Note amplitude and note rate increases markedly along the first half of the call, decreasing again at the end. The call has two harmonics, the first harmonic has slightly more energy than the second (Fig. 6). Fundamental frequency of individual notes ranges from 404.3 to 585.9 Hz (mean = 500.08, SD = 28.3). The dominant frequency of the entire call is 515.6 Hz. Midway through the call, nearly 25% of the notes have the greatest energy in the second harmonic (range 1016.6 to 1210 Hz).

##### Distribution and natural history.

*Tepuihyla shushupe* is only known from the type locality in the headwaters of rivers Ere and Campuya, at an elevation of 145 m, in the Putumayo river basin near the boundary between Peru and Colombia. According with Vriesendorp (2013), the type locality consists of a complex of forest terraces, at elevations between 90 and 170 m above sea level, with a canopy reaching 35 to 40 m; terraces have heavy loads of leaf litter (~50 cm deep) and a dense mat of fine roots; the depressions between terraces have small palm swamps (~10 m wide) of *Oenocarpus bataua*; the soil varies between sandy and clayey; most streams have a muddy bottom, few have gravel and sand, and one has big cobbles.

The holotype was calling at the base of a big tree inside a narrow hole, 150 cm above the ground. The hole had 30 cm of height and had water accumulated. The frog had most of its body submerged (Fig. 10C) and the top of its head was covered by flies (probably *Corethrella* midges). We recorded its call and immediately made playbacks. The male answered by calling quickly and perching on the hole entrance. We detected at least six individuals during 18 hours of visual encounter surveys (0.375 individuals/hour,) in areas nearby where the holotype was found (primary forest). All individuals were detected by their advertisement calls and none could be collected. No females, amplectant pairs, clutches, or tadpoles have been observed.

##### Etymology.

The specific epithet is a noun in apposition. The word *shushupe* is used by native people to refer to the bushmaster *Lachesis muta* (Squamata: Viperidae), the largest viper in the Americas. Our field assistants in Ere river, Alpahuayo Mishana (Peru) and Juyuintza (Ecuador) believed that the advertisement calls of *Tepuihyla shushupe* and *Tepuihyla tuberculosa* were produced by *Lachesis muta*. The belief that *Lachesis muta* can sing seems to be widespread among hunters, colonists, and indigenous people from the Amazon basin (Lamar 1998). The association of the calls from *Tepuihyla* with *Lachesis muta* by people on widely separated localities in Amazonian Peru and Ecuador deserves investigation.

##### Conservation status.

*Tepuihyla shushupe* is only known from a single individual collected at the type locality. Calling behavior suggests that *Tepuihyla shushupe* breeds on tree holes and is a canopy dweller. The detection of the calls of six additional individuals at the type locality suggests that the species can be relatively abundant. However, the species may be difficult to observe in ground-level surveys. Given the uncertainty in its population status, we suggest that its Red List category is Data Deficient according to IUCN (2001) guidelines.

##### Remarks.

The juvenile specimen (USNM 193866) from Rio Querari (Amazonas, Brazil) reported as *Ecnomiohyla tuberculosa* by Duellman (1974) probably correspond to *Tepuihyla shushupe*, but a taxonomic validation is needed to confirm its identity.

### Taxonomic status of populations of *Trachycephalus typhonius* from Ecuador and Peru

Several authors have discussed the considerable morphological variation in *Trachycephalus typhonius* (Duellman 1971; McDiarmid 1968; Savage 2002) and have suggested that it may represent a species complex (Cochran and Goin 1970; Duellman 2005). The paraphyly of *Trachycephalus typhonius* (Fig. 1A) also suggests the existence of undescribed species. The resolution of the taxonomy of *Trachycephalus typhonius* is challenging given the large number of available synonyms (Duellman 1971; Lavilla et al. 2010). However, the identification of the type locality of *Trachycephalus typhonius* as Paramaribo, Suriname, by Lavilla et al. (2010) allows inferring the phylogenetic position of *Trachycephalus typhonius* *sensu stricto*. The type locality is geographically close to samples from Guyana (AMNH-A 141142) and French Guiana (163MC), Clade C in Figure 1A. Therefore, we consider Clade C to represent *Trachycephalus typhonius* *sensu stricto*. Two linages from Ecuador and Peru are distinct from Clade C and each has an available name. Below we discuss their status.

### “*Trachycephalus typhonius*” from the lowlands of western Ecuador

**Figure 11.**
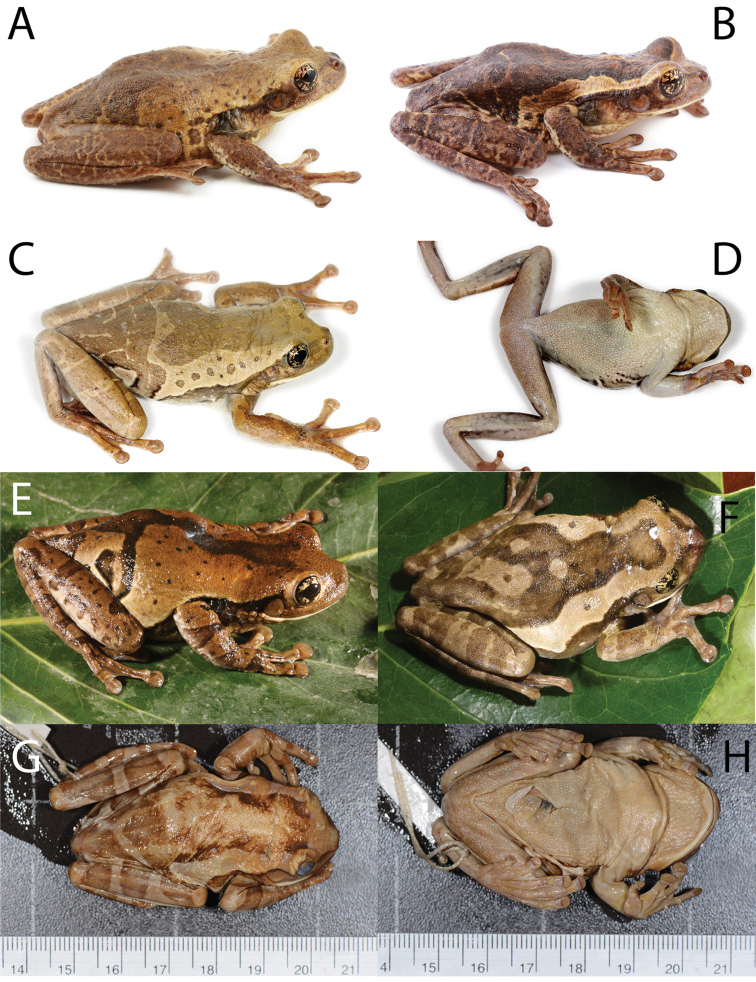
External morphology of *Trachycephalus quadrangulum* comb. n. from the Chocó region, western Ecuador. **A** QCAZ 39360, adult male, SVL = 74.1 mm, Caimito, Provincia de Esmeraldas, Ecuador **B** QCAZ 39361, adult male, SVL = 73.2 mm, same locality as A **C–D** QCAZ 51746, adult female, SVL = 80.3, Bosque Pacocha, Provincia Manabí, Ecuador **E** QCAZ 35406, adult male (SVL = 69.5), Bosque Protector Puyango, Provincia El Oro, Ecuador **F** QCAZ 28530, adult male (SVL = 64.5), Hacienda Santa Teresita, Provincia del Guayas, Ecuador; (**G–H**) Holotype of *Hyla quadrangulum*, adult female; scale, in cm, shown for reference. Photographs: **A–F** by S. R. Ron, **G–H** by Jeff Streicher.

Populations from western Ecuador are sister to a clade composed of *Trachycephalus cunauaru*, *Trachycephalus hadroceps*, *Trachycephalus resinifictrix* and *Trachycephalus typhonius*. Their phylogenetic position demonstrates that they are not conspecific with *Trachycephalus typhonius* *sensu stricto* or other species of *Trachycephalus*. One binomial available for *Trachycephalus* from western Ecuador is *Hyla quadrangulum* Boulenger 1882 (type locality “western Ecuador”). Duellman (1971) synonymized *Hyla quadrangulum* under *Trachycephalus coriaceus* stating that the holotype is “indistinguishable from *Phrynohyas coriacea*”. He also questioned its type locality because “several species contained in the Fraser collections supposedly from western Ecuador have been found subsequently only in the Amazon Basin”. Our examination of a large series of specimens from western Ecuador and *Trachycephalus coriaceus* from the Amazon basin show unequivocally that the holotype of *Hyla quadrangulum* (Fig. 11G–H) is not conspecific with *Trachycephalus coriaceus*. Although the holotype has a quadrangular dorsal blotch, also present in *Trachycephalus coriaceus*, we found the same pattern in specimens from western Ecuador (e.g., QCAZ 23427, 31302, 39361, 51746). Moreover, the holotype lacks the dark blue blotch above the arm characteristic of *Trachycephalus coriaceus*. We conclude that the type locality is correct and *Hyla quadrangulum* is a valid species. The resurrection of *Hyla quadrangulum* Boulenger 1882 results in *Trachycephalus quadrangulum* (Boulenger 1882) comb. n.

Names under *Trachycephalus typhonius* that could be senior synonyms to *Trachycephalus quadrangulum* include *Hyla spilomma* Cope 1877 (type locality Veracruz, México), *Hyla paenulata* Brocchi 1879 (“versant occidental du Guatemala”), and *Hyla nigropunctata* Boulenger 1882 (several localities in Mexico). We exclude from this list synonyms with type localities east of the Andes based on our phylogeny, which shows that *Trachycephalus quadrangulum* is restricted to the Chocó region. The phylogeny is consistent with a general biogeographic pattern showing that humid lowlands east and west from the Andes do not share amphibian species as result of the vicariant effect of the Andes (dos Santos et al. 2015). The phylogenetic and biogeographic evidence indicates that it is highly unlikely that available binomials with type localities in the Amazon basin or Guianan region are conspecific with *Trachycephalus quadrangulum*. The resolution of the taxonomic identity of populations from Central America and the Chocó region is beyond the scope of this article. Nevertheless, the resurrection of *Trachycephalus quadrangulum* provides a name to an evolutionary lineage separate from *Trachycephalus typhonius*. Even if provisional, this new arrangement better reflects the evolutionary uniqueness of Chocoan populations and contributes to a more accurate characterization of the species diversity of the Chocoan amphibiofauna. It also improves our understanding of the conservation status of the *Trachycephalus typhonius* species complex.

### “*Trachycephalus typhonius*” from Amazonian Ecuador and Peru

**Figure 12.**
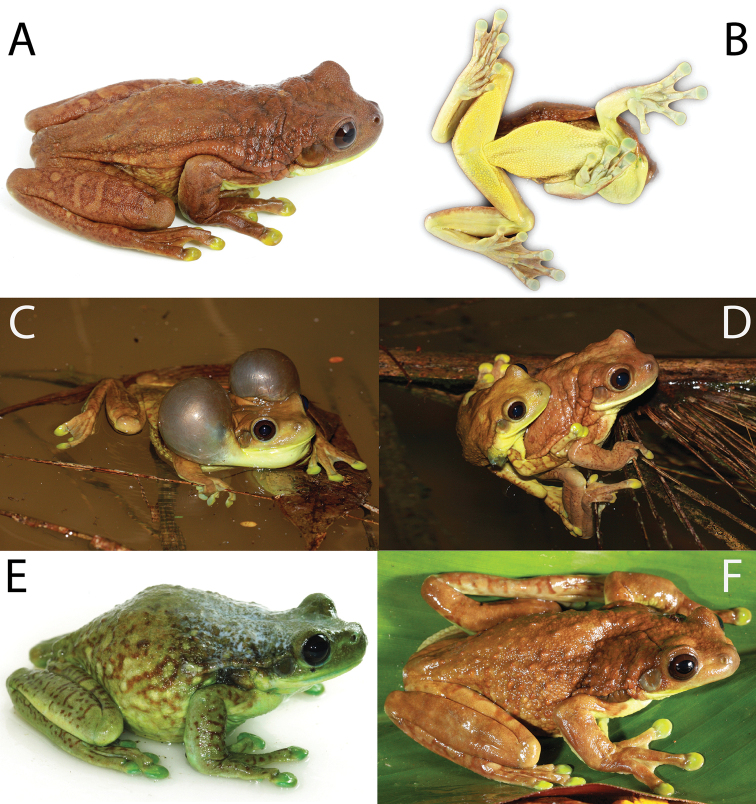
External morphology of *Trachycephalus macrotis* comb. n. **A–B** QCAZ 39565, adult female, SVL = 101.1 mm, Reserva Zanjarajuno, Ecuador **C** calling male, Reserva Zanjarajuno, Ecuador **D** amplectant pair, QCAZ 39565, adult female (SVL = 101.12) and QCAZ 39566, adult male (SVL = 81.38), Reserva Zanjarajuno, Ecuador **E** CORBIDI 9544, adult male, (SVL = 82.9), Peru **F** QCAZ 43017, adult male, SVL = 91.5 mm, Parque Nacional Yasuní, km 22 Pompeya-Iro road, Ecuador. Photographs by S. R. Ron except for (**E**) by A. Catenazzi.

According to the phylogeny (Fig. 1A), populations from Amazonian Ecuador and Peru (Clade B) are sister to *Trachycephalus resinifictrix* and this clade is sister to *Trachycephalus typhonius* *sensu stricto*. Clade B is morphologically distinct from *Trachycephalus resinifictrix* and comparisons of live individuals of clade B with photographs of the holotype of *Trachycephalus typhonius* published by Lavilla et al. (2010) reveal that they belong to separate species as well. The holotype of *Trachycephalus typhonius* (UUZM 134) shows the clear iris with dark reticulations, also characteristic of populations from French Guyana (Fig. 8D–F). Clade B, in contrast, is characterized by having a dark brown iris without reticulations (Fig. 12).

**Figure 13.**
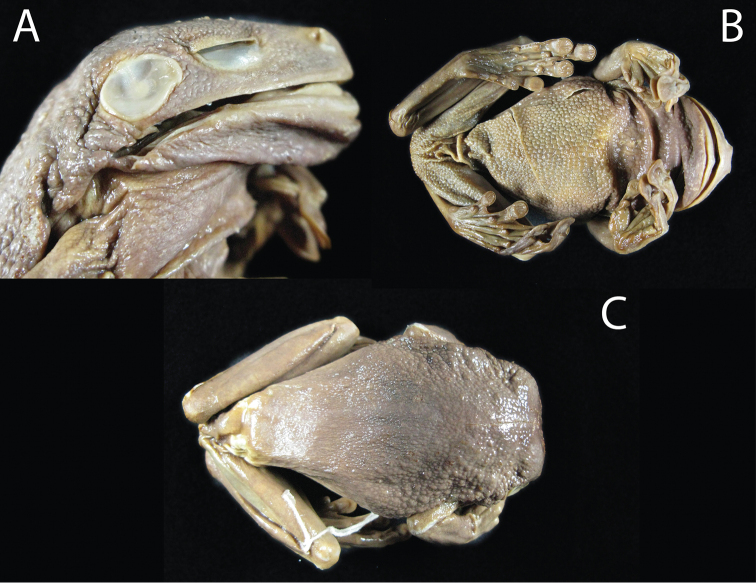
Holotype of *Hyla macrotis* Anderson 1945, NHRM 1958. **A** lateral view of the head **B** ventral view **C** dorsal view. The holotype is an adult female, SVL = 120 mm (Anderson 1945). Photographs by Bo Delling.

Given its phylogenetic position and distinct external morphology, we conclude that Clade B is not conspecific with *Trachycephalus typhonius*. We propose a solution for the status of those populations by resurrecting the binomial *Hyla macrotis* Andersson 1945 from its synonymy under *Trachycephalus typhonius* proposed by Duellman (1971). The resurrection is based in the similarity between the holotype of *Hyla macrotis* (Fig. 13) and specimens from Clade B (Fig. 12). Within the region where the holotype was collected, “Río Pastaza watershed, Ecuador”, only clade B is morphologically similar. This allows us to confidently conclude that the holotype of *Hyla macrotis* and clade B are conspecific. The resurrection of *Hyla macrotis* results in *Trachycephalus macrotis* (Andersson 1945) comb. n.

It seems unlikely that other synonyms under *Trachycephalus typhonius* are senior synonyms to *Trachycephalus macrotis* because most of them are from the Guianan region and Central America and are geographically distant from the range of *Trachycephalus macrotis*. The resurrection of *Trachycephalus macrotis* provides a name to an evolutionary lineage separate from *Trachycephalus typhonius*. As in *Trachycephalus quadrangulum*, the recognition of *Trachycephalus macrotis* better reflects the biology of its populations.

We could not examine the voucher specimens for the GenBank sequences of *Trachycephalus* used in our phylogeny. Some of those identifications could be incorrect and should be revaluated. For example, specimen KU 217753 is identified as *Trachycephalus venulosus* (= *Trachycephalus typhonius*) from the Chocó region of Ecuador. However, in the phylogeny it is nested within *Trachycephalus cunauaru* from Amazonian Ecuador. Both the identification and locality are incongruent with its phylogenetic position suggesting that the tissue actually belongs to *Trachycephalus cunauaru* from the Amazon region. The same error applies to GenBank sequence DQ347027, a sample that was obtained from the pet market. GenBank sequence JQ868532 (field number PS013) is identified as *Trachycephalus venulosus* (= *Trachycephalus typhonius*). The specimen is likely lost and its correct identification appears to be *Trachycephalus resinifictrix*. Despite these potential identification errors, our taxonomic conclusions are only dependent on the correct identifications of the samples of *Trachycephalus typhonius* from Guyana and French Guyana, which are reliable. We obtained photographs of populations from French Guyana (Fig. 8) from the same collector who provided the tissues for individual 163MC. Therefore, the identification of the tissue should be correct. Sequences for the specimen from Guyana were published by Faivovich et al. (2005). They have a voucher specimen (AMNHA 121142), which should be correctly identified.

### Morphological comparisons between *Trachycephalus quadrangulum* and *Trachycephalus macrotis*

The external morphology of *Trachycephalus quadrangulum* is distinct from that of *Trachycephalus macrotis*. Populations from western Ecuador (Fig. 1; Fig. 11) are smaller (adult males average SVL = 69.0 mm, range 53.4–76.9, n = 9; adult females SVL = 75.5 mm, 60.5–80.8 mm; n = 10; Ron and Read 2011), have brown to cream disks on the fingers, and a bronze iris with irregular black reticulations (Fig. 11); *Trachycephalus macrotis* (clade B, Fig. 1) are larger (adult males average SVL = 84.58 mm, range 69.8–91.5, n = 12; adult females SVL = 103.2 mm, 93.9–118.7, n = 4), have green discs on the fingers, and a dark brown iris (Fig. 12). The morphologic and genetic differences between both clades corroborate that they represent separate species.

## Discussion

These results show, for the first time, the evolutionary affinities of *Tepuihyla tuberculosa*, a species of controversial position within Hylidae (Faivovich et al. 2005; Savage and Kubicki 2010). The inclusion of *Tepuihyla tuberculosa* within the clade *Tepuihyla* was unexpected because it has a highly divergent morphology compared to other congeners. *Tepuihyla tuberculosa* and *Tepuihyla shushupe* differ from all other *Tepuihyla* in having tuberculate skin texture in the dorsum, extensive webbing between the fingers and toes, serrate fringes on limbs, and calling behavior from tree-holes (Ayarzagüena et al. 1992; Duellman and Hoogmoed 1992; Gorzula and Señaris 1998; Kok and Kalamandeen 2008; Mijares-Urrutia et al. 1999). Except for size, all these characters are putative synapomorphies for *Tepuihyla tuberculosa* and *Tepuihyla shushupe* because they are absent in other *Tepuihyla* and its sister clade, *Dryaderces* + *Osteocephalus*. The distinctiveness of *Tepuihyla tuberculosa* and *Tepuihyla shushupe* is remarkable considering that both *Tepuihyla* and *Osteocephalus* lack phenotypic synapomorphies except for juvenile color pattern in *Osteocephalus* (Jungfer et al. 2013). Phytotelmata breeding, in contrast, is shared between all *Tepuihyla* and could be a synapomorphy for the genus or for a larger clade that also includes *Osteocephalus* and *Trachycephalus* because both genera have species that breed in phytotelmata (e.g., Gordo et al. 2013; Jungfer et al. 2000).

The morphological similarity between *Tepuihyla tuberculosa* and *Ecnomiohyla* could be the result of convergence resulting from a similar reproductive mode and microhabitat. *Ecnomiohyla* are large frogs that call and lay eggs on tree holes partly filled with water (McCranie et al. 2003; Mendelson et al. 2008; Savage 2002; Savage and Kubicki 2010). Our observations of males of *Tepuihyla tuberculosa* and *Tepuihyla shushupe* calling from tree holes containing water suggest they share a similar reproductive mode. Convergence in external morphology and reproductive mode in *Ecnomiohyla* and Amazonian *Tepuihyla* suggests that both sets of characters are correlated. This hypothesis is reinforced by the morphological similarity between *Tepuihyla tuberculosa* and *Trachycephalus cunauaru*, *Trachycephalus hadroceps* and *Trachycephalus resinifictrix* (Fig. 8). These three species of *Trachycephalus* are among the few Amazonian hylids that also call and breed on tree holes (Duellman and Trueb 1976; Gordo et al. 2013; Moser 2010). They represent an additional independent origin of this mode of reproduction. As in *Ecnomiohyla*, *Tepuihyla tuberculosa*, and *Tepuihyla shushupe*, large size, mossy coloration, ornamented skin, and large hands characterize their external morphology.

The phylogenetic position of *Tepuihyla tuberculosa* is consistent with the biogeography of Hylinae. Most species of the tribe Hylini (including *Ecnomiohyla*) occur in Central and North America; only *Ecnomiohyla phantasmagoria* and three species of *Smilisca* are present in South America and none of them occur in the Amazon basin (Wiens et al. 2006). According to the biogeographic reconstruction by Wiens et al. (2011), the most recent common ancestor (MRCA) of Hylini inhabited either Central América or North America while the MRCA of *Ecnomiohyla* inhabited Central America. Therefore, the assignment of “*Hyla tuberculosa*” to the genus *Ecnomiohyla* was at odds with the distribution of the rest of the tribe. The tribe Lophiohylini, on the other hand, is primarily distributed in South America. The MRCA of *Tepuihyla* probably occurred in the Guyana region, the MRCA of *Osteocephalus* in the Amazon basin and that of *Tepuihyla* + *Osteocephalus* + *Dryaderces* in Guyana-Amazon or Amazon basin exclusively (Wiens et al. 2011). The phylogenetic relationships of *Tepuihyla tuberculosa* reported hare imply a more parsimonious biogeographic scenario because they do not require a recent dispersal event between Central America and the Amazon Basin. Further analyses with a more complete taxon sampling are needed to more confidently infer the geographic origin of *Tepuihyla*.

## Supplementary Material

XML Treatment for Tepuihyla tuberculosa

XML Treatment for Tepuihyla shushupe

## Supplementary materials

Supplementary material 1 Majority rule consensus for maximum likelihood phylogenetic analysis based on Pyron (2014) matrix for genes 12S and 16S rRNA with additional sequences of Amazonian *Tepuihyla* Data type: Phylogeny Explanation note: The consensus was obtained from 100 non-parametric bootstrap pseudoreplicates. Bootstrap Support values are shown on nodes. This dataset is made available under the Open Database License (http://opendatacommons.org/licenses/odbl/1.0/). The Open Database License (ODbL) is a license agreement intended to allow users to freely share, modify, and use this Dataset while maintaining this same freedom for others, provided that the original source and author(s) are credited.Santiago R. Ron, Pablo J. Venegas, H. Mauricio Ortega-Andrade, Giussepe Gagliardi-Urrutia, Patricia Salerno

Supplementary material 2 Examined material Data type: Specimen and occurence data Explanation note: Specimens are deposited in the following institutions. Ecuador: Fundación Herpetológica Gustavo Orcés, Quito (FHGO); Museo de Zoología at Pontificia Universidad Católica del Ecuador, Quito (QCAZ). Peru: Centro de Ornitología y Biodiversidad, Lima (CORBIDI); Colección Referencial de Biodiversidad del Instituto de Investigaciones de la Amazonía Peruana, Iquitos (IIAP). United States of America: American Museum of Natural History, New York, (AMNH); National Museum of Natural History, Washington DC (USNM); Natural History Museum at the University of Kansas, Lawrence (KU). United Kingdom: Natural History Museum, London (BMNH). Venezuela: Museo de Historia Natural La Salle, Caracas (MHNLS). This dataset is made available under the Open Database License (http://opendatacommons.org/licenses/odbl/1.0/). The Open Database License (ODbL) is a license agreement intended to allow users to freely share, modify, and use this Dataset while maintaining this same freedom for others, provided that the original source and author(s) are credited.Santiago R. Ron, Pablo J. Venegas, H. Mauricio Ortega-Andrade, Giussepe Gagliardi-Urrutia, Patricia Salerno
