# Supplementary figures and images for: Systematics of Ecnomiohyla tuberculosa with the description of a new species and comments on the taxonomy of Trachycephalus typhonius (Anura, Hylidae)
Source: Zookeys. 2016 Nov 9;(630):115–54. doi: 10.3897/zookeys.630.9298 (PMC5126525; doi:10.3897/zookeys.630.9298)

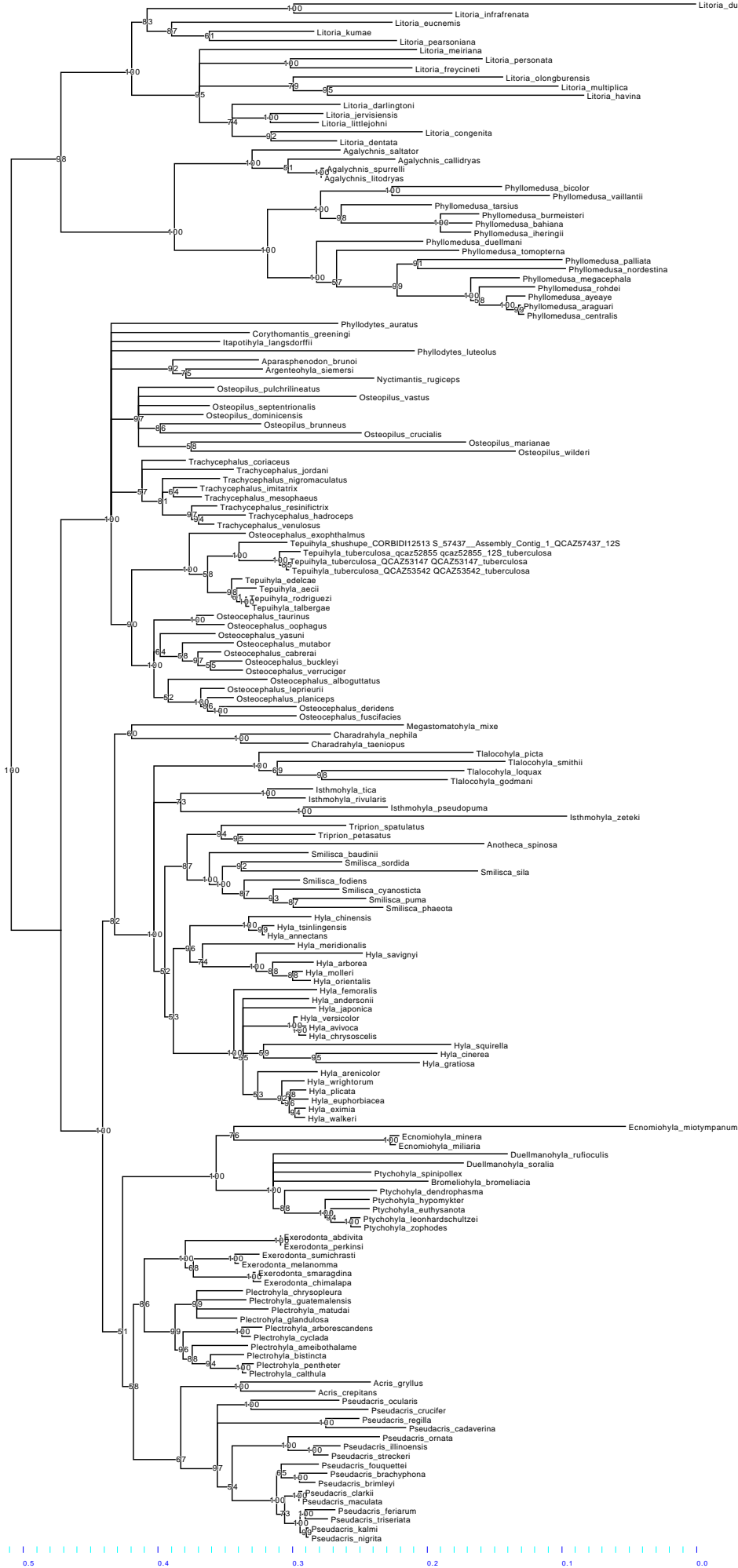

Supplement: Supplementary material 1 — Majority rule consensus for maximum likelihood phylogenetic analysis based on Pyron (2014) matrix for genes 12S and 16S rRNA with additional sequences of Amazonian Tepuihyla [file zookeys-630-115-s001.pdf]
